# Diverging trends in aerosol sulfate and nitrate measured in the remote North Atlantic in Barbados are attributed to clean air policies, African smoke, and anthropogenic emissions

**DOI:** 10.5194/acp-24-8049-2024

**Published:** 2024-07-16

**Authors:** Cassandra J. Gaston, Joseph M. Prospero, Kristen Foley, Havala O. T. Pye, Lillian Custals, Edmund Blades, Peter Sealy, James A. Christie

**Affiliations:** 1 Rosenstiel School of Marine, Atmospheric, and Earth Science, University of Miami, Miami, FL 33149, USA; 2 Office of Research and Development, U.S. Environmental Protection Agency, Research Triangle Park, North Carolina, USA

## Abstract

Sulfate and nitrate aerosols degrade air quality, modulate radiative forcing and the hydrological cycle, and affect biogeochemical cycles, yet their global cycles are poorly understood. Here, we examined trends in 21 years of aerosol measurements made at Ragged Point, Barbados, the easternmost promontory on the island located in the eastern Caribbean Basin. Though the site has historically been used to characterize African dust transport, here we focused on changes in nitrate and non-sea-salt (nss) sulfate aerosols from 1990–2011. Nitrate aerosol concentrations averaged over the entire period were stable at 0.59 μg m^−3^ ± 0.04 μg m^−3^, except for elevated nitrate concentrations in the spring of 2010 and during the summer and fall of 2008 due to the transport of biomass burning emissions from both northern and southern Africa to our site. In contrast, from 1990 to 2000, nss-sulfate decreased 30% at a rate of 0.023 μg m^−3^ yr^−1^, a trend which we attribute to air quality policies enacted in the United States (US) and Europe. From 2000–2011, sulfate gradually increased at a rate of 0.021 μg m^−3^ yr^−1^ to pre-1990s levels of 0.90 μg m^−3^. We used the Community Multiscale Air Quality (CMAQ) model simulations from the EPA’s Air QUAlity TimE Series (EQUATES) to better understand the changes in nss-sulfate after 2000. The model simulations estimate that increases in anthropogenic emissions from Africa explain the increase in nss-sulfate observed in Barbados. Our results highlight the need to better constrain emissions from developing countries and to assess their impact on aerosol burdens in remote source regions.

## 1 Introduction

Sulfate and nitrate aerosols, formed from gaseous sulfur dioxide (${\text{SO}}_{2}$), nitrogen oxides (e.g., ${\text{NO}}_{x}\equiv \text{NO}+{\text{NO}}_{2}$), and reactive nitrogen (e.g., ${\text{NO}}_{y}$), contribute to aerosol direct and indirect radiative forcing, impact biogeochemical cycles (Jickells et al., 2017), and degrade air quality (Adams et al., 1999; Appel et al., 1978; Charlson et al., 1992; Lelieveld and Heintzenberg, 1992; Murphy et al., 2006). An outstanding question is, how have sulfate and nitrate aerosol burdens in remote regions changed in response to air quality policies, economic growth, and changing frequency of wildfires – all of which have affected ${\text{SO}}_{2}$ and ${\text{NO}}_{x}$ emissions? Answering this question is important, as remote regions are an important barometer of changing global emission inventories, contain ecosystems sensitive to changing chemical inputs (Galloway et al., 2008; Mahowald et al., 2017), and are most sensitive to fluctuations in aerosol burdens that alter aerosol–cloud interactions (Carslaw et al., 2013). Long-term measurement records in remote regions can provide insight into this question and further advance current chemical transport and global climate models. However, there are few long-term measurement records in remote regions. In this work, we leverage 21 years of nitrate and sulfate aerosol concentrations measured at Ragged Point, Barbados, a remote site in the eastern North Atlantic marine boundary layer, and use simulations from a hemispheric chemical transport model – the Community Multiscale Air Quality (CMAQ) model within the EPA’s Air QUAlity TimE Series (EQUATES) (Foley et al., 2023) – to link our observed changes in nitrate and sulfate to changing emissions inventories and meteorological conditions. In turn, comparing the EQUATES model output to our time series provides guidance on where in situ measurements are needed to improve emissions inventories and measurement–model agreement.

Ragged Point, Barbados, provides a unique opportunity to understand changes in the nitrate and sulfate aerosol burden in the remote North Atlantic marine boundary layer. Aerosol sampling began in 1971 and continues to this day, generating a 50-year measurement record – the longest modern speciated aerosol record, to the best of our knowledge (Prospero et al., 2021; Prospero and Mayol-Bracero, 2013). The site serves as a linchpin for understanding the impact of long-range aerosol transport on the remote North Atlantic marine boundary layer and the Caribbean. The site’s primary objective has been to understand the factors affecting the long-range transport of African dust to the Caribbean and North America, which peaks in boreal summer in association with the seasonal northward shift in the intertropical convergence zone (ITCZ). Summer dust events are caused by the strong heating of northern Africa, which causes hot, dry, dust-laden desert air to be carried to high altitudes, e.g., 4–6 km. African easterly waves propagate dust westward within an elevated air layer known as the Saharan Air Layer (SAL) that overrides the cool, moist marine boundary layer (Adams et al., 2012; Carlson and Prospero, 1972; Goudie and Middleton, 2001; Tsamalis et al., 2013). Background emissions at the site are dominated by sea spray and marine biogenic emissions of dimethyl sulfide (DMS) that contribute non-sea-salt (nss) sulfate (Andreae et al., 1985; Barnes et al., 2006; Savoie et al., 2002). Along with dust, anthropogenic emissions from Europe (Lelieveld et al., 2002), North America, and northern Africa are also transported to Barbados (Savoie et al., 2002). Transport from Africa takes ~5–7 d to reach our site, while transport from the United States (US) and Europe takes longer, typically 7–10 d.

Anthropogenic emissions of ${\text{SO}}_{2}$ and ${\text{NO}}_{x}$ that impact Ragged Point have changed in recent decades due to the opposing effects of decreasing emissions mandated by national air quality policies, implemented mostly in developed countries, and increasing emissions linked to rapid economic growth in developing countries. The US curbed emissions of ${\text{NO}}_{x}$ and ${\text{SO}}_{2}$ with the implementation of the Clean Air Act (amended in 1990), resulting in a 92% reduction in ${\text{SO}}_{2}$ and a 71% reduction in ${\text{NO}}_{x}$ emissions from 1990–2022 (Aas et al., 2019; Hand et al., 2012; https://gispub.epa.gov/air/trendsreport/2023/, EPA, 2023a; Smith et al., 2011; Zhao et al., 2017). Countries within the European Union (EU) passed similar policies resulting in analogous reductions (Aas et al., 2019; Rafaj et al., 2015; Smith et al., 2011; Yang et al., 2020). Notably, reductions in ${\text{SO}}_{2}$ can reduce aerosol acidity, resulting in increased nitrate aerosols. Furthermore, reductions in pollutant gases can relieve oxidant limitations, leading to more efficient oxidation; therefore, reductions in ${\text{SO}}_{2}$ and ${\text{NO}}_{x}$ may not reduce sulfate and nitrate aerosols as much as expected (Shah et al., 2018). In contrast to the US and EU, emissions in regions such as the Middle East, India, and Africa are continuing to increase due to rapid economic growth, with emissions from India predicted to overtake China as the world’s largest emitter of ${\text{SO}}_{2}$ (Lelieveld et al., 2009; Li et al., 2017; McDuffie et al., 2020). Due to a lack of in situ measurements in many of these regions, chemical transport and emissions inventory models combined with remote sensing have been key tools to understand changing pollutants.

In addition to fossil fuel emissions, biomass burning is also a major source of ${\text{SO}}_{2}$ and ${\text{NO}}_{x}$ that can impact the Atlantic (Andreae, 2019; Andreae and Merlet, 2001; Rickly et al., 2022; Roberts et al., 2009; Zuidema et al., 2018). Wildfire activity has a distinct seasonality linked to the dry seasons in Africa. Burning is most intense in sub-Saharan Africa from the Equator to 20° N from November through May, while, from May through October, the savanna regions of sub-Saharan Africa from the Equator to 25° S are the most active fire sources (Giglio et al., 2006; Kganyago and Shikwambana, 2019; Roberts et al., 2009; Van der Werf et al., 2003). African smoke can be transported to Barbados from sub-Saharan Africa north of the Equator in winter and spring (Quinn et al., 2022; Royer et al., 2023; Wex et al., 2016) and, less frequently, from southern hemispheric Africa in the fall (Trapp et al., 2010). Conditions thought to be related to African climate (e.g., the North Atlantic Oscillation and the position of the Azores High) can cause large quantities of northern African dust (and smoke) to be transported during the winter and spring in elevated mixed aerosol layers (Chiapello et al., 2005; Doherty et al., 2008, 2012; Gutleben et al., 2022) when dust is also carried to northeastern South America (Barkley et al., 2019; Prospero et al., 1981, 2014). Prescribed burns in the southeastern US in winter and spring may also contribute biomass burning emissions to the aerosol burden observed in Barbados (Nowell et al., 2018).

Here we highlight different trends in nitrate and sulfate aerosols over the remote North Atlantic marine boundary layer and relate them to changing emissions. We then compare our observations to simulated concentrations of nitrate and sulfate aerosols using the CMAQ model from EQUATES, which was chosen due to its skill in modeling changes in nitrate and sulfate chemistry within the US (Benish et al., 2022) and its ability to simulate constituents and sources of air pollution in remote regions, such as Dhaka, Bangladesh (Sarwar et al., 2023). Our results highlight the importance of long-term atmospheric observations to understand the net outcome of changing global ${\text{SO}}_{2}$ and ${\text{NO}}_{x}$ emissions on both the aerosol burden and the air quality in distant populations.

## 2 Methods

### 2.1 Aerosol collection at the Barbados Atmospheric Chemistry Observatory

**Figure 1.**
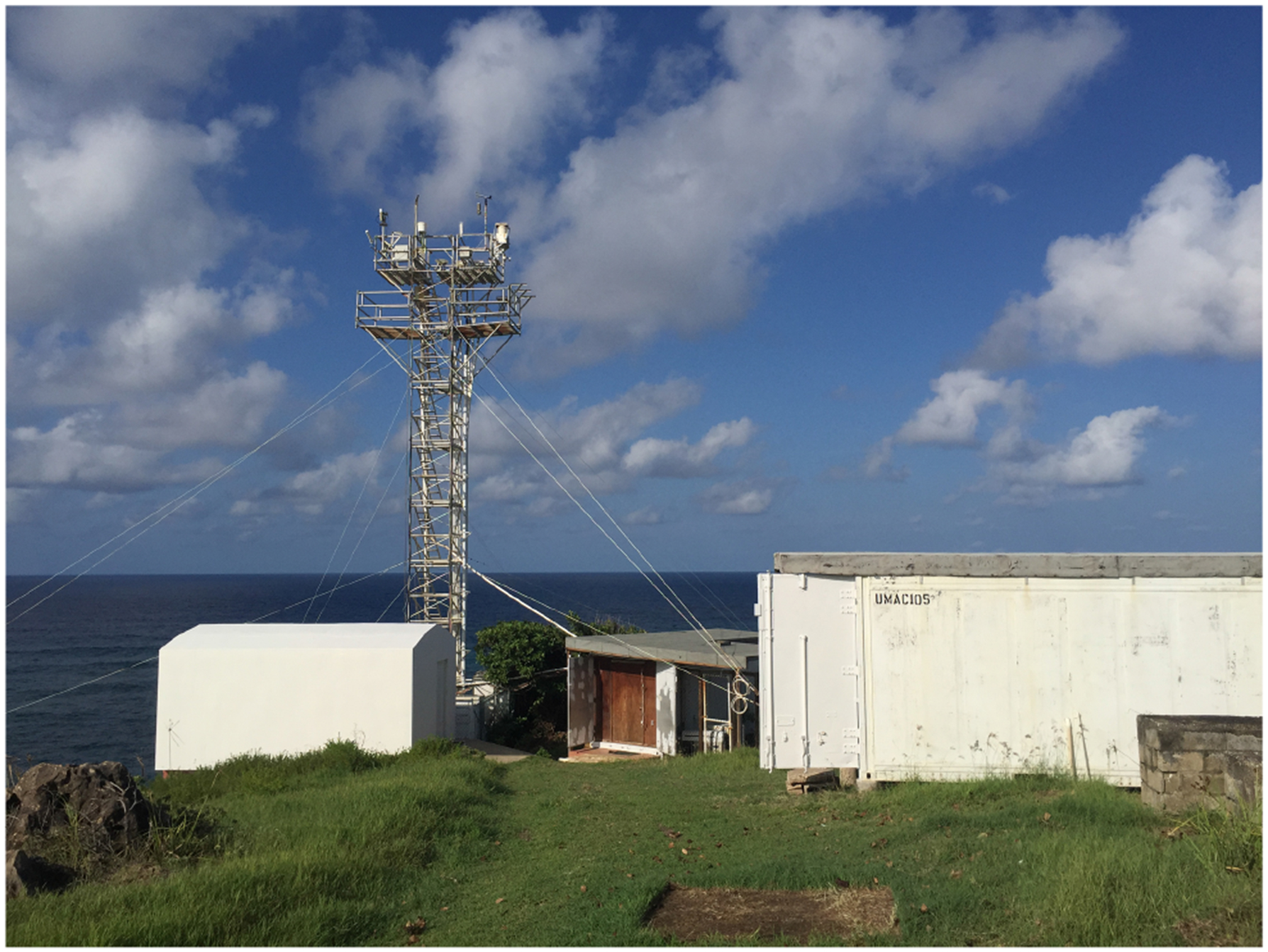
Photo of the Barbados Atmospheric Chemistry Observatory (BACO), including the 17 m sampling tower, two shipping container laboratories, and an Advanced Global Atmospheric Gases Experiment (AGAGE) laboratory.

Aerosols were collected daily at the University of Miami’s Barbados Atmospheric Chemistry Observatory (UM BACO: https://baco.rsmas.miami.edu/, last access: 10 July 2024) located at Ragged Point, Barbados – the easternmost promontory on the east coast of the island (13.16504° N, 59.43207° W). The site has been operated by UM since 1971, and aerosol data have been used to document the long-range transport of African dust to the Caribbean and the Americas carried by the easterly trade winds, as it is the first land-mass encountered by African emissions transported across the Atlantic Ocean (Prospero et al., 2021). The site is approximately 4500 km from the west coast of northern Africa, 3000 km from the east coast of the US, and 6000 km from the west coast of the EU. Since 1989, aerosols have been collected at the top of a 17 m sampling tower that stands atop a 30 m bluff (see Fig. 1).

A high-volume sampler pulled air at a nominal rate of 0.75 m^3^ min^−1^ across a 20 cm × 25 cm Whatman 41 filter. The upper particle size limit for our filter collection method was approximately 80–100 μm or greater based on the geometry of our sampling rain hat (Barkley et al., 2021). Filters were collected daily (e.g., every 24 h); however, a few multiday samples that typically spanned 2 d were also collected. Mass collection efficiencies were 90% for sulfate, 95% for nitrate, and 99% for dust (Savoie and Prospero, 1982). Filters were then folded into quarters under a laminar flow hood, placed in a clean Ziploc bag, and periodically shipped to UM for processing. To ensure that local island emissions were not sampled, the sampling pump was only operational when the wind blew directly from the ocean (from 335 degrees through N and E to 130 degrees) with speeds greater than 1 m s^−1^. A timer was used to record the “run time”, the total amount of time that the sampling pumps were on during the sampling interval between filter changes. Data with a run time less than 10% of the sampling interval were discarded to minimize the impact of low-speed and flukey wind conditions that might have carried aerosols from local sources. These deletions accounted for less than 10% of all data collected over the record, highlighting the steady easterly winds measured at the site year-round. Procedural blanks were collected weekly by placing a Whatman 41 filter in the filter cassette with the sampling pump off for 15 min then placing the filter in a clean Ziploc bag; the blank was subsequently processed along with the daily filter samples.

This work focuses on aerosol measurements conducted over 1990–2011, a period that overlaps with the implementation of more stringent air quality policies in the US and Europe. Seasonal trends are also shown, where winter is represented by December (from the previous year), January, and February (DJF); spring is March, April, and May (MAM); summer is June, July, and August (JJA); and fall is September, October, and November (SON). African dust peaks annually at BACO in JJA with episodic transport in DJF and MAM in some years (Prospero and Mayol-Bracero, 2013; Quinn et al., 2022; Royer et al., 2023). Trends presented in this work are derived from Theil–Sen regression. Significance is calculated using paired-sample *t* tests.

### 2.2 Quantification of dust and soluble ion mass concentrations:

A one-quarter filter section was extracted 3 times with a total volume of 20 mL of Milli-Q water to remove soluble material. The extracted filter was placed in a combustion oven (500 °C) overnight. The resulting ash was weighed ($m_{\text{filter ash}}$) and subsequently corrected for the filter background by subtracting the ash measured from performing the same technique on the procedural blank ($m_{\text{procedural blank}}$). The gross ash weight was adjusted by a factor of 1.3 to account for losses during the extraction and combustion process (Prospero, 1999; Zuidema et al., 2019). This corrected ash mass concentration was equated to mineral dust concentrations present on the filter based on previous comparisons between filter ash and concentrations of aluminum, a tracer for mineral dust (Prospero, 1999; Trapp et al., 2010).


(1) $$\text{dust}=(m_{\text{filter}\;\text{ash}}-m_{\text{procedural blank ash}})\cdot 1.3$$

The filtrate from the sample extraction process and procedural blanks was used to quantify soluble ion concentrations. Anions (e.g., chloride (${\text{Cl}}^{-}$), nitrate (${\text{NO}}_{3}^{-}$), and sulfate (${\text{SO}}_{4}^{2-}$)) were measured using ion chromatography (IC). Cations (sodium (${\text{Na}}^{+}$), potassium ($K^{+}$), and calcium (${\text{Ca}}^{2+}$)) were measured with a flame photometer after 2004, while flame atomic absorption spectrophotometry was used prior to 2004, limiting cation analysis to sodium (Savoie et al., 2002). In addition to total soluble ion concentrations, we also report concentrations of non-sea-salt (nss) sulfate, which is a useful tracer of sulfur from marine biogenic and pollution emissions, and nss-potassium, a tracer of biomass burning emissions (Andreae, 1983; Keene et al., 1986). Concentrations of $\text{nss}-{\text{SO}}_{4}^{2-}$ and $\text{nss}-K^{+}$ were calculated using the following mass-based equations and assuming that ${\text{Na}}^{+}$ is a conservative tracer of sea spray aerosols.


(2) $$\text{nss}-{\text{SO}}_{4}^{2-}=\left[{\text{SO}}_{4}^{2-}\right]-(0.2517\cdot [{\text{Na}}^{+}])$$

(3) $$\text{nss}-K^{+}=[K^{+}]-(0.03595\cdot [{\text{Na}}^{+}])$$

Filter samples with undetectable amounts of dust and soluble ions compared to procedural blanks are removed from our analysis.

### 2.3 EQUATES model products

EPA’s Air QUAlity TimE Series (EQUATES) project uses the Community Multiscale Air Quality (CMAQ) model, a 3-D chemical transport air quality model, to simulate air quality over a continuous 2002–2019 period (Foley et al., 2023). CMAQ accounts for gas, cloud, and aerosol chemistry, including processes such as in-cloud sulfate formation from the oxidation of ${\text{SO}}_{2}$. EQUATES uses CMAQv5.3.2 (Appel et al., 2021) to model the Northern Hemisphere using 108 km resolution and 44 vertical layers (Mathur et al., 2017). Meteorological data are derived from the Weather Research and Forecasting model (WRFv.4.1.1). Emissions from outside the contiguous US and China are generated using the Hemispheric Transport of Air Pollution version 2 (HTAPv2.2) inventory for the year 2010 and are scaled to other years using the Community Emissions Data System (CEDS) for the years 2002–2019. The Fire INventory from NCAR (FINN) is used to generate biomass burning emissions (Wiedinmyer et al., 2011), lightning ${\text{NO}}_{x}$ emissions are derived from the Global Emissions InitiAtive (GEIA), biogenic volatile organic compounds (VOCs) are from MEGAN2, and soil ${\text{NO}}_{x}$ is from CAMSv2.1.

In this study, EQUATES was used to better understand observed trends, namely in nss-sulfate, after 2000 when observed concentrations unexpectedly increased. EQUATES also simulated concentrations of locally emitted gases, including ${\text{SO}}_{2}(g)$ and ${\text{NO}}_{x}(g)$, which are not measured at Ragged Point. The model product is not available for the years prior to 2000. The analysis focused on dust, sea spray, nitrate, sulfate, and gaseous ${\text{SO}}_{2}$ and ${\text{NO}}_{2}$. In addition to anthropogenic sources of ${\text{SO}}_{2}$, natural sources from the oxidation of DMS were included in model runs. Species predictions were extracted from the lowest CMAQ model layer (~ 10 m in thickness) for a source area over the Atlantic Ocean to the east of the island from 14.3989 to 11.45667° N latitude and 59.5627 to 56.54487° W longitude (equivalent to 16 grid cells with 1 cell over Ragged Point and the others to the east of the site). Simulated concentrations of aerosol sulfate, nitrate, calcium, potassium, and sodium were obtained for fine-mode aerosols (e.g., Aitken and accumulation mode, PMF model outputs) and coarse-mode aerosols (total PM–PM_2.5_, PMC model outputs). Previous studies have shown that most of the aerosol mass at Ragged Point is below 10 μm diameter (Prospero et al., 2001). Because aerosol filters collected at BACO capture total suspended particulate matter, model outputs of fine- and coarse-mode aerosol concentrations were combined (e.g., PMF + PMC model outputs) to give total aerosol mass concentrations of sulfate, nitrate, sodium, potassium, and calcium. Total concentrations of nss-sulfate and nss-potassium were calculated using Eqs. (2) and (3), respectively, and model outputs of total sodium mass concentrations, total sulfate mass concentrations, and total potassium mass concentrations were calculated from combined coarse- and fine-mode aerosol model outputs. We calculated concentrations of nss-calcium, which has been shown to be a good tracer for mineral dust in Barbados (Savoie and Prospero, 1980), and dust mass concentrations were then calculated using the average upper-crustal abundance of calcium in soil (an average of 4.1 %) (Scheuvens et al., 2013; Taylor and McLennan, 1985) as shown in Eqs. (4) and (5).


(4) $$\text{nss}-{\text{Ca}}^{2+}=\left[{\text{Ca}}^{2+}\right]-(0.0376\cdot [{\text{Na}}^{+}])$$

(5) $$\text{dust}=\left[\text{nss}-{\text{Ca}}^{2+}\right]\cdot 24.1$$

We first assessed the ability of the EQUATES CMAQ simulations to capture trends in different aerosol types observed at Ragged Point. Simulations of sodium and dust from EQUATES (see Figs. S1 and S2 in the Supplement) capture seasonal and monthly observed trends in dust. However, the model overpredicts sodium (${\text{Na}}^{+}$, a proxy for sea spray) by a factor of 3–4 (Figs. S1) and underpredicts dust by an average factor of ~ 7. This low bias for dust in CMAQv5.3.2 is consistent with CMAQ development that occurred after the EQUATES simulations were complete. A bug fix to the online dust emissions module in CMAQv5.4 increases dust emissions by a factor of 3–7 over the Sahara and parts of Asia (see the CMAQv5.4 release notes for further information; https://www.epa.gov/cmaq/cmaq-documentation#release-notes, last access: 10 July 2024).

In addition to simulating observed aerosol concentrations, EQUATES was also used to examine trends in gaseous indicators of anthropogenic and biomass burning emissions, oxidant concentrations and oxidant ratios important for investigating changes in the oxidation efficiency of locally emitted pollutant gases, and the subsequent formation of nitrate and sulfate aerosols. Furthermore, EQUATES was used to investigate whether the oxidation efficiency of ${\text{SO}}_{2}(g)$ changed during the 2002–2011 period. The oxidation ratio was calculated from Eq. (6) (Shah et al., 2018):

(6) $$\text{oxidation ratio}=\frac{\text{nss}-{\text{SO}}_{4}^{2-}}{\text{nss}-{\text{SO}}_{4}^{2-}+{\text{SO}}_{2}}.$$

For this calculation, we used EQUATES model data for ${\text{SO}}_{2}(g)$ concentrations and filter-based observations of nss-sulfate.

### 2.4 HYSPLIT back-trajectory analysis

Air mass back-trajectory analysis was performed using NOAA’s Hybrid Single-Particle Lagrangian Integrated Trajectory (HYSPLIT) model (Draxler and Rolph, 2011; Rolph et al., 2017; Stein et al., 2015). Back-trajectories of 13 d were initiated at heights of 500, 1000, and 2000 m to capture both SAL and boundary layer transport. Frequency plots were also generated for the entire sampling period (1990–2011) to illustrate seasonal differences in air mass transport and origin and to explore any interannual variability. We used the global National Centers for Environmental Prediction (NCEP) re-analysis data that extend back to the beginning of our dataset in 1990 (Kramer et al., 2020). The finer-resolution Global Data Assimilation System (GDAS) dataset (1° resolution) was used to examine trends in air mass back-trajectories in 2008, 2009, and 2010 when transport conditions strongly impacted nitrate aerosol concentrations. We emphasize the challenges in characterizing long-range-transported aerosols due to the remoteness of the site, the location of Barbados in the middle of the ocean, and the fact that air layers of smoke and dust change in height during transport. As such, we caution against the over-reliance on HYSPLIT analysis to extensively characterize long-range transport aerosol dynamics and instead use this analysis to quantitatively describe major changes in transport patterns.

## 3 Results

### 3.1 Measured trends from 1990–2011 at Ragged Point

**Figure 2.**
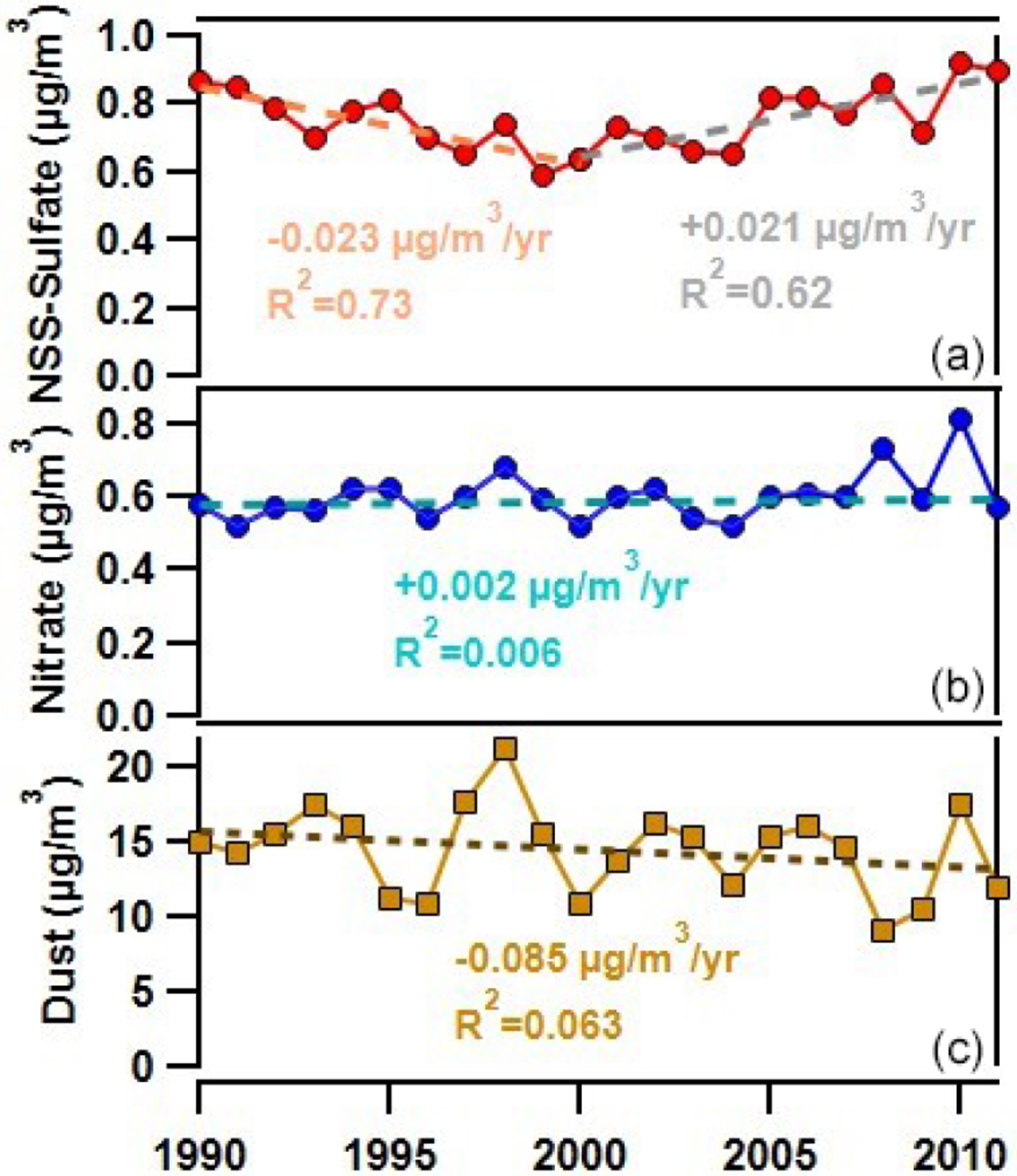
Yearly averages of **(a)** non-sea-salt sulfate (nss-sulfate; red line), **(b)** nitrate (blue line), and **(c)** dust (brown line) measured at Ragged Point, Barbados, from 1990–2011. Dashed lines show changes in nss-sulfate pre-2000 (orange) and post-2000 (gray), nitrate (teal) without the spikes in 2008 and 2010 considered, and dust (brown) over the 1990–2011 period.

Figure 2 shows yearly averaged mass concentrations of non-sea-salt (nss) sulfate and nitrate from 1990 through 2011. Nitrate concentrations are remarkably stable from 1990 to 2011 (*R*^2^ = 0.006, *p* > 0.05 (not significant)) with an average concentration of 0.59 μg m^−3^ ± 0.04 μg m^−3^. However, two anomalous peaks in nitrate are observed in 2008 and 2010 with annual average nitrate concentrations of 0.73 and 0.81 μg m^−3^, respectively. Similarly, dust mass concentrations also show no trend over this period (*R*^2^ = 0.06). In contrast, nss-sulfate decreases by 30 % from an average concentration of 0.84 μg m^−3^ starting in 1990 to a minimum of 0.64 μg m^−3^ in 2000 at a rate of −0.023 μg m^−3^ yr^−1^. Subsequently, sulfate gradually increased to 0.91 μg m^−3^ in 2010 and 0.90 μg m^−3^ in 2011, maximums across the entire record. The trends in the yearly average mass concentrations of nitrate and nss-sulfate are significantly different (*p* value < 0.005), which can be explained either by different sources or different rates of change for precursor ${\text{NO}}_{x}$ and ${\text{SO}}_{2}$ emissions or by different responses in nitrate and sulfate aerosol production to changing emissions of ${\text{NO}}_{x}$ and ${\text{SO}}_{2}$, as has been shown in North America (Shah et al., 2018; Vasilakos et al., 2018).

The Barbados yearly concentrations differ from long-term observations of aerosol and precipitation chemistry measured at Tudor Hill, a site on the west coast of Bermuda, from 1989–1997 and from 2006–2009 as part of the same program as that in Barbados (AEROCE) and using the same protocols including sampling only when winds are over the ocean (Keene et al., 2014; Savoie et al., 2002). At the Bermuda site, the prevailing winds come from the west so that the sampling sector extends from 180° through west to 330°. Aerosol nitrate is constant at both sites. However, the nitrate annual mean in Bermuda is ~ 1.05 μg m^−3^, roughly double the nitrate observed at Ragged Point (Keene et al., 2014; Savoie et al., 2002). Also, the decline in nss-sulfate observed in Bermuda, from ~ 2.59 μg m^−3^ in 1989 to ~ 1.63 μg m^−3^ in 2009, is greater than our observations at Ragged Point (Keene et al., 2014). Furthermore, sulfate aerosols in Bermuda decline over the entire record and do not exhibit the same reversal in the 2000s that we observe in Barbados. The differences in concentration and trend in nss-sulfate observed at Ragged Point compared to in Bermuda are not surprising given that the Bermuda site is located 1100 km from the east coast of the US and is more directly influenced by anthropogenic emissions compared to Barbados, which is more remote and could be influenced by a multitude of emission sources (Savoie et al., 2002). Also, the trend in nss-sulfate in Barbados differs from long-term observations measured at an IMPROVE site in the US Virgin Islands. Sulfate shows no trend from 2004–2021 (Hand et al., 2024). The difference in trend in nss-sulfate between Ragged Point and the IMPROVE site in the US Virgin Islands is likely due to more influence from the US and less influence from African emissions. Furthermore, the IMPROVE site does not follow a sector-controlled sampling protocol as the site in Barbados does.

To assess whether annual trends in nss-sulfate and nitrate observed in Barbados are associated with African aerosol transport conditions, annual average measured dust mass concentrations are also shown in Fig. 2 and show no appreciable increase or decrease from 1990–2011 (*R*^2^ = 0.06). Therefore, annual trends in nss-sulfate aerosols, which show a decrease from 1990–2000 and an increase from 2000–2011, are not correlated with African dust mass concentrations (*R*^2^ = 0.001). This suggests that the increase in nss-sulfate after the year 2000 is not due to an increase in sulfate associated with heterogeneous reactions between ${\text{SO}}_{2}(g)$ and dust or to more favorable transport from Africa. Nitrate aerosols, in contrast, are modestly correlated with dust (*R*^2^ = 0.30). Comparing seasonal nitrate and dust mass concentrations by year reveals tighter correlations between nitrate and dust for DJF and MAM (0.46 and 0.4, respectively; see Fig. S3). DJF and MAM are not the peak dust transport seasons to the Caribbean, but they are the seasons that favor co-transport of dust and biomass burning emissions from sub-Saharan Africa north of the Equator (Royer et al., 2023). Transport of African smoke to Barbados has been shown to be associated with elevated concentrations of nitrate, which likely explains the association between winter- and springtime dust and nitrate (Quinn et al., 2021; Savoie and Prospero, 1982).

#### 3.1.1 Seasonal patterns in air mass trajectories and nitrate concentrations

HYSPLIT air mass back-trajectories reveal similar seasonal patterns each year with predominantly easterly transport to the site year-round (see Fig. S4, which shows 5 d air mass back-trajectory frequency plots for the entire 1990–2011 period and similar patterns year to year). As has been documented in prior work (Prospero et al., 2021), the dominant transport pathways are over the ocean and traverse the African continent. Some trajectories do intercept North America and come close to Europe, while few (< 10%) air masses come near South America, which is outside of our sampling sector. Air masses typically take 5–7 d to be transported from northern Africa and longer than 7 d to intercept the EU and North America. According to Fig. S4, DJF has some trajectories that intercept North America and some from the northern part of the Atlantic Ocean toward Europe, MAM trajectories are easterly, JJA trajectories are from the west coast of Africa, and SON trajectories are from the east (with some from the southeast). We note that the directional tendencies of the trajectories did not noticeably change in 2000, when the concentration trends in nss-sulfate changed.

**Figure 3.**
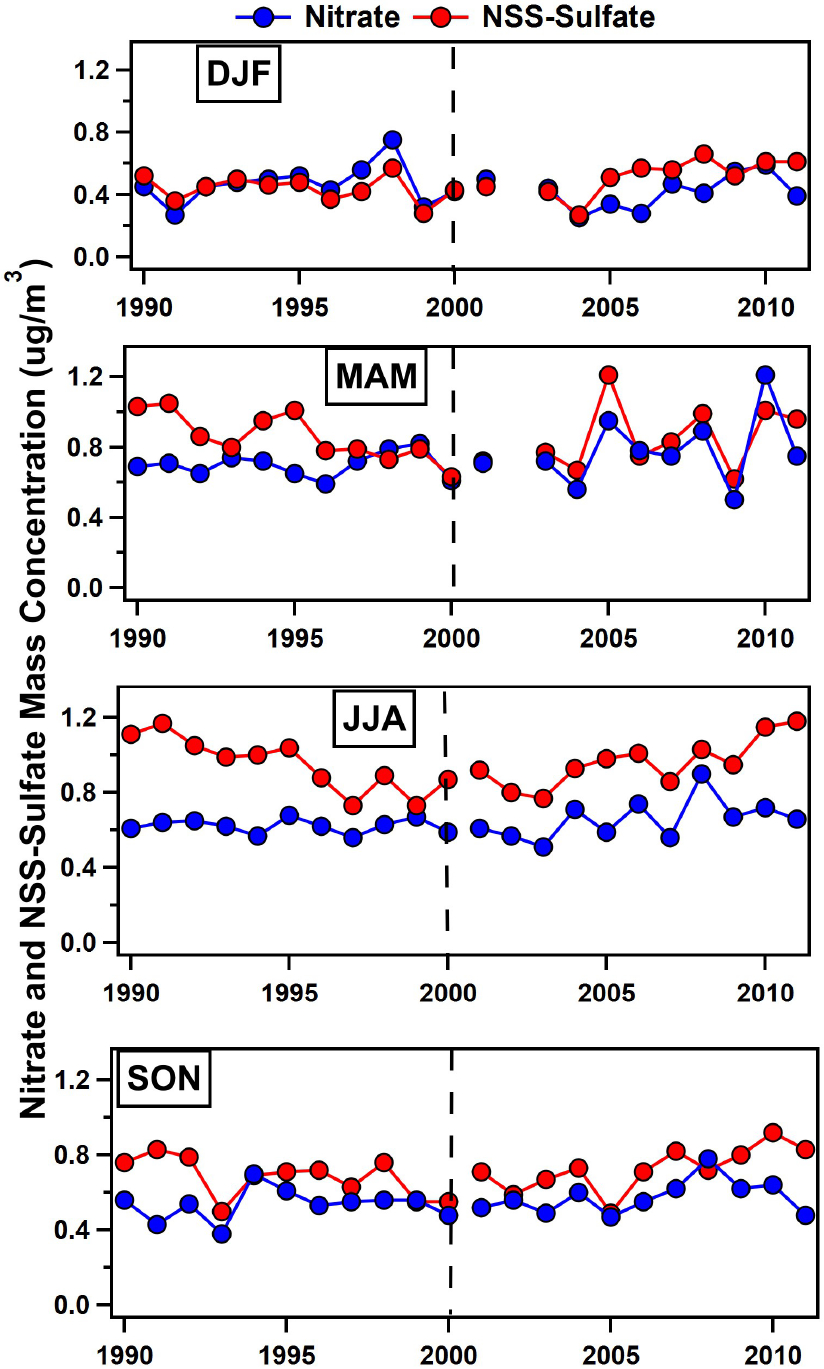
Average concentrations of nitrate (blue markers) and nss-sulfate (red markers) for winter (DJF), spring (MAM), summer (JJA), and fall (SON). The vertical dashed black line denotes the year 2000 when nss-sulfate trends shifted from decreasing to increasing.

**Figure 4.**
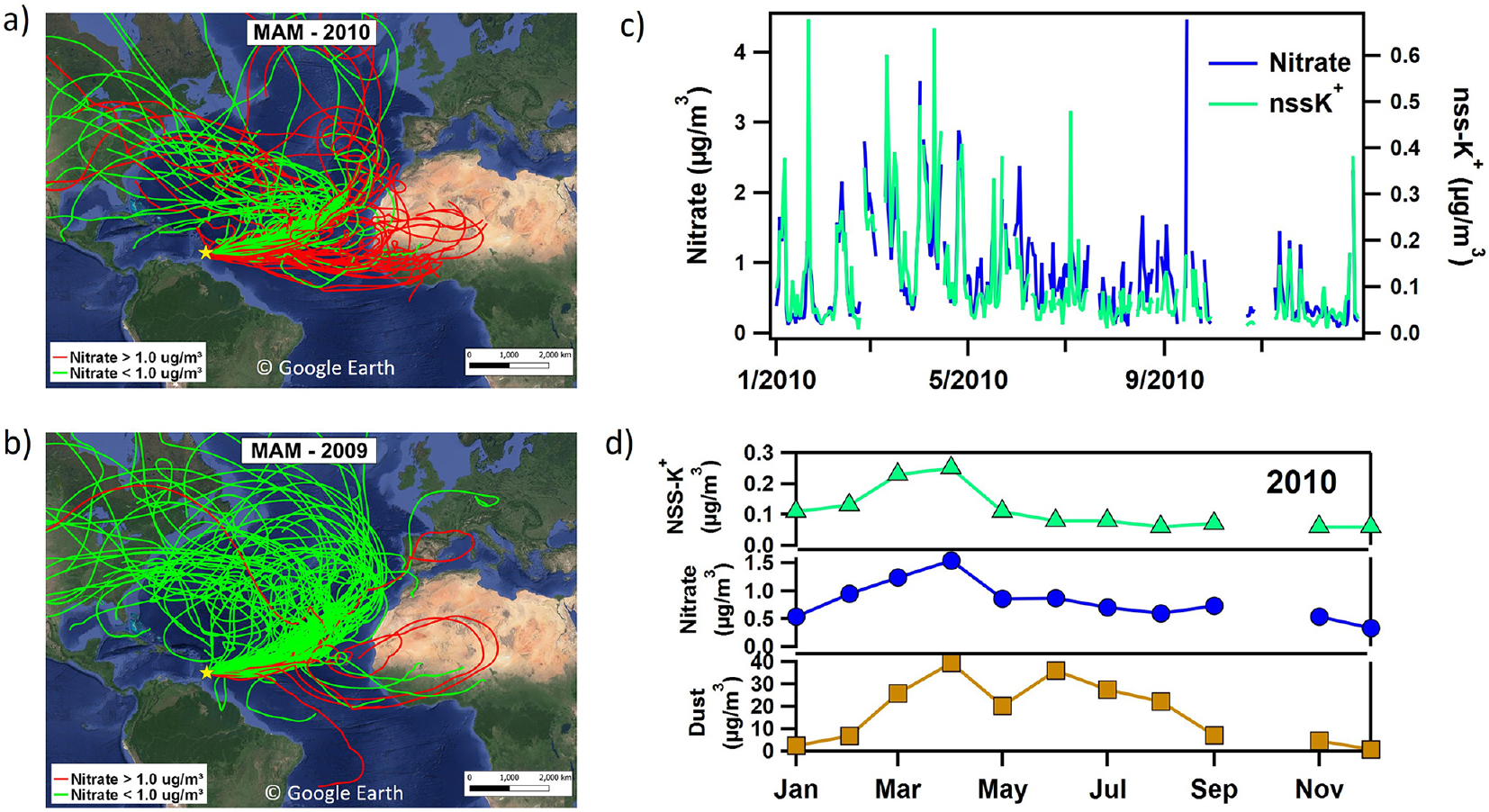
13 d air mass back-trajectories initiated at 500 m for MAM 2010 **(a)** and 2009 **(b)**. Back-trajectories labeled in green are for days where nitrate concentrations measured at Ragged Point were < 1 μg m^−3^, while trajectories labeled in red had nitrate > 1 μg m^−3^. Maps are from Google Earth (©Google Earth). **(c)** Daily comparison of nitrate and non-sea-salt potassium ($\text{nss}-K^{+}$), a biomass burning marker, shows a tight correlation (*R*^2^ = 0.62). **(d)** Monthly average concentrations of dust (brown), nitrate (blue), and $\text{nss}-K^{+}$ (green) for 2010.

Figure 3 shows trends in nss-sulfate and nitrate during different seasons (e.g., DJF, MAM, JJA, and SON). Increases in nitrate in 2008 are driven by high nitrate concentrations in JJA and SON, with most of the increase in September, while high nitrate levels in 2010 are primarily observed during MAM. In 2010, a transition from El Nino to the strongest La Niña event on record occurred (Wolter and Timlin, 2011; Zhang et al., 2019), and long-range transport from Africa was anomalously high during the springtime as evidenced by high mass concentrations of dust in the spring of 2010 (Zuidema et al., 2019). Daily air mass back-trajectories passed more frequently over the African continent in MAM of 2010 compared to MAM of 2009 (26 vs. 6 d) (Fig. 4a and b), and nitrate levels exceeded 1 μg m^−3^ on over half of those days when trajectories traversed the northern African continent. Figure 4 focuses on trajectories initiated at 500 m height. If trajectories initiated at 1000 m are also included, then transport over the northern African continent occurs on 36 d in MAM of 2010; i.e., on 82% of the days, nitrate levels exceeded 1 μg m^−3^. However, during the summertime peak (JJA) in African dust transport in 2010, nitrate does not show the same increase as dust despite frequent transport from northern Africa (see Fig. 4d). Furthermore, Fig. 4c and d compare daily and monthly mean concentrations of nitrate, dust, and non-sea-salt potassium (e.g., $\text{nss}-K^{+}$), which is a marker for biomass burning emissions (Andreae and Merlet, 2001). Nitrate and $\text{nss}-K^{+}$ clearly track each other and are both elevated in the spring of 2010. Although biomass burning peaks in December and January, while MAM is the tail end of the burning season in northern sub-Saharan Africa (Giglio et al., 2013; Roberts et al., 2009), the high nitrate loadings observed in spring 2010 are likely due to strongly favorable transport conditions that transported both dust and smoke to Barbados during this year.

**Figure 5.**
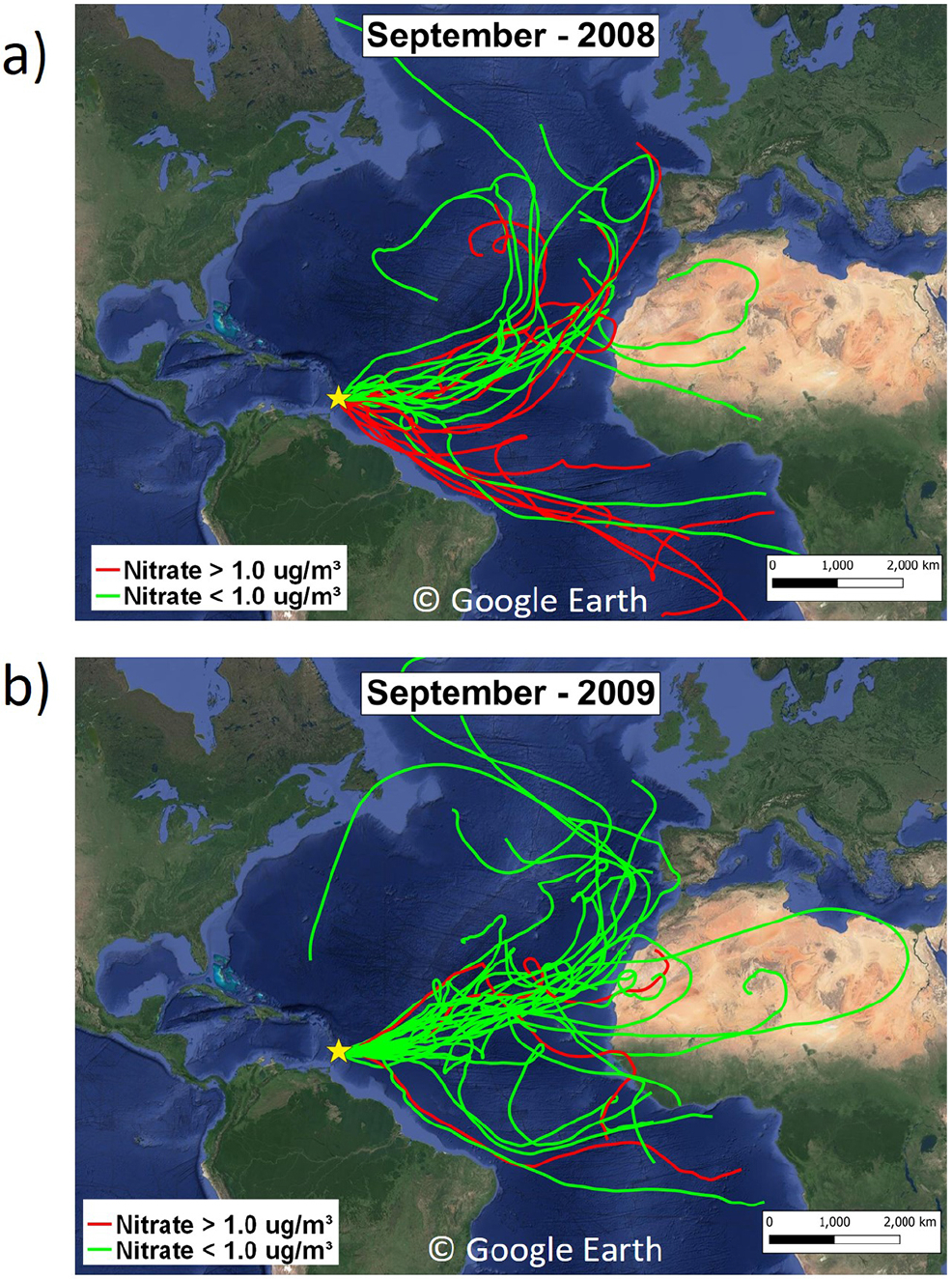
Air mass back-trajectories of 13 d initiated at 500 m for September 2008 **(a)** and 2009 **(b)**. Back-trajectories labeled in green are for days when nitrate concentrations measured at Ragged Point were < 1 μg m^−3^, while trajectories labeled in red had nitrate > 1 μg m^−3^. The maps are from Google Earth (©Google Earth).

Similarly to 2010, air mass back-trajectories traversed the African continent on just 40% of the days in JJA but 82% of the days in September of 2008 with nitrate concentrations > μg m^−3^ (Figs. S5 and 5a). If trajectories initiated at the 1000 m level are included, then African transport occurs on 64% of the days in JJA with nitrate concentrations > 1 μg m^−3^, consistent with higher-level transport in JJA. In JJA, most of the air mass back-trajectories pass through Africa north of the Equator. However, in September, trajectories take a more southerly route, traversing near the South American coast and sub-Saharan Africa south of the Equator (Fig. 5a). Figure 5b shows trajectories for September but in 2009, when fewer trajectories take a southerly route and nitrate concentrations rarely exceed 1 μg m^−3^. September is during the peak of the burning season in sub-Saharan Africa south of the Equator and also dovetails with strong smoke transport over the Atlantic Ocean due to the increased intensity of the African easterly jet (Adams et al., 2012; Adebiyi and Zuidema, 2016; Zuidema et al., 2018). Air mass back-trajectories in September from sub-Saharan Africa south of the Equator to our measurement sites in Barbados and Cayenne have been linked with African smoke transport (Barkley et al., 2019; Trapp et al., 2010), further suggesting a link between elevated concentrations of nitrate measured in Barbados with transported African smoke. Biomass burning in the Amazon also peaks in SON (Adams et al., 2012) and could also explain some of the increase in nitrate in this season in 2008.

#### 3.1.2 Trends in non-sea-salt sulfate and emissions of ${\text{SO}}_{2}$

Figure 3 reveals that the decrease in sulfate and subsequent increase are measured during most seasons. Table 1 further provides correlation coefficients and the rate of change in either nitrate or sulfate in μg m^−3^ yr^−1^ for data collected pre- and post-2000 when the trend in nss-sulfate changed. Pre-2000, nss-sulfate shows a consistent decline in each season, with the weakest decline in winter and the strongest reduction measured in JJA at −0.036 μg m^−3^ yr^−1^ (*R*^2^ = 0.72; Table 1). Post-2000, nss-sulfate increased during every season, with comparable increases in every season. In JJA, nss-sulfate increased at +0.028 μg m^−3^ yr^−1^ (*R*^2^ = 0.61; Table 1). In contrast, nitrate shows no trend (e.g., *R*^2^ < 0.2) in any season except a slight increase of +0.012 μg m^−3^ yr^−1^ post-2000 in JJA. Unlike nitrate, which showed intermittent spikes associated with increased biomass burning aerosol transport periods, the persistent increases in nss-sulfate observed during all seasons more likely reflect either increased emissions or more efficient oxidation of ${\text{SO}}_{2}$.

**Figure 6.**
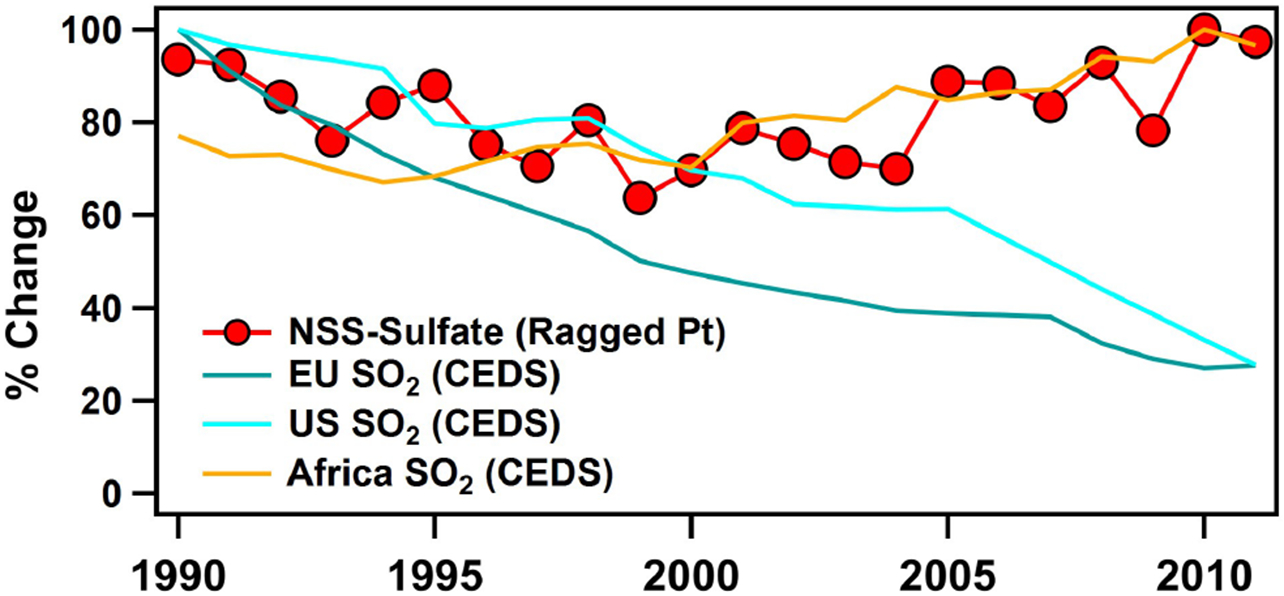
Percent changes in nss-sulfate mass concentrations measured at Ragged Point from 1990–2011 are shown in red. nss-sulfate mass decreases from 0.87 μg m^−3^ in 1990 to 0.64 μg m^−3^ in 2000 and subsequently increases to 0.90 μg m^−3^ in 2011. The data are normalized to the maximum nss-sulfate mass concentration, 0.92 μg m^−3^, measured in 2010. The percent changes in emissions of sulfur dioxide (${\text{SO}}_{2}$) from the CEDS emissions inventory from McDuffie et al. (2020) are included for comparison. Decreasing emissions of ${\text{SO}}_{2}$ from the US and EU are shown with blue lines. US ${\text{SO}}_{2}$ reduces from 21.12 to 5.85 Tg S yr^−1^, and EU ${\text{SO}}_{2}$ reduces from 28.06 to 7.74 Tg S yr^−1^. Percentages are calculated by normalizing to the maximum values in ${\text{SO}}_{2}$ emissions observed in 1990 for both the US and EU. ${\text{SO}}_{2}$ emissions from Africa increased from 4.13 to 5.95 Tg S yr^−1^; they are normalized to the maximum ${\text{SO}}_{2}$ emissions observed in 2010 at 6.16 Tg S yr^−1^.

Figure 6 compares yearly trends in Ragged Point nss-sulfate along with ${\text{SO}}_{2}$ emissions reported from the Community Emissions Data System (CEDS) (McDuffie et al., 2020). We focus on the most likely sources to impact Ragged Point: the US, the EU, and Africa. A similar figure comparing nitrate concentrations measured at Ragged Point and nitrogen dioxide (${\text{NO}}_{2}$) emissions from CEDS can be found in the Supplement (Fig. S6). Our near-constant nitrate mass concentrations do not match the decline in ${\text{NO}}_{2}$ observed in the EU and US and the increase in ${\text{NO}}_{2}$ in Africa. In contrast, decreases in nss-sulfate observed from 1990–2000 at Ragged Point closely follow the 32% and 58% reductions in ${\text{SO}}_{2}$ emissions in the US and Europe, respectively (Fig. 6) (Aas et al., 2019; Hand et al., 2012; McDuffie et al., 2020; Rafaj et al., 2015; Yang et al., 2020). Our finding that changing ${\text{SO}}_{2}$ emissions in the EU and US are reflected in our record at Ragged Point agrees with previous work examining both anthropogenic and biogenic sulfate (Savoie et al., 2002). Figure 6 also compares the trends of nss-sulfate observed at Ragged Point to increasing emissions of ${\text{SO}}_{2}$ from Africa (McDuffie et al., 2020). Before 2000, ${\text{SO}}_{2}$ emissions from Africa oscillated around 4.44 ± 0.19 Tg S yr^−1^ but show no consistent trend (*R*^2^ = 0.027). However, after 2000, emissions of ${\text{SO}}_{2}$ from Africa steadily increased by 37% from 2000–2011 (4.33 Tg S yr^−1^ in 2000 and 5.95 Tg S yr^−1^ in 2011; *R*^2^ = 0.88). The rate of increase in ${\text{SO}}_{2}$ is on par with the rate of increase in nss-sulfate of 29% observed in Barbados, suggesting that anthropogenic emissions from Africa are affecting the nss-sulfate trends measured in Barbados.

We next utilized the CMAQ model results from EQUATES to gain further insight into the observed recovery of nss-sulfate (post-2000). We first note that EQUATES also predicts an increase in ${\text{SO}}_{2}$ emissions from northern hemispheric Africa after 2002 (see Fig. S7). However, we use EQUATES to determine if other factors, such as changes in the oxidation efficiency of locally emitted ${\text{SO}}_{2}$ and meteorological changes, affected our observations.

### 3.2 Comparison of measured and modeled trends of nitrate and sulfate aerosols

**Figure 7.**
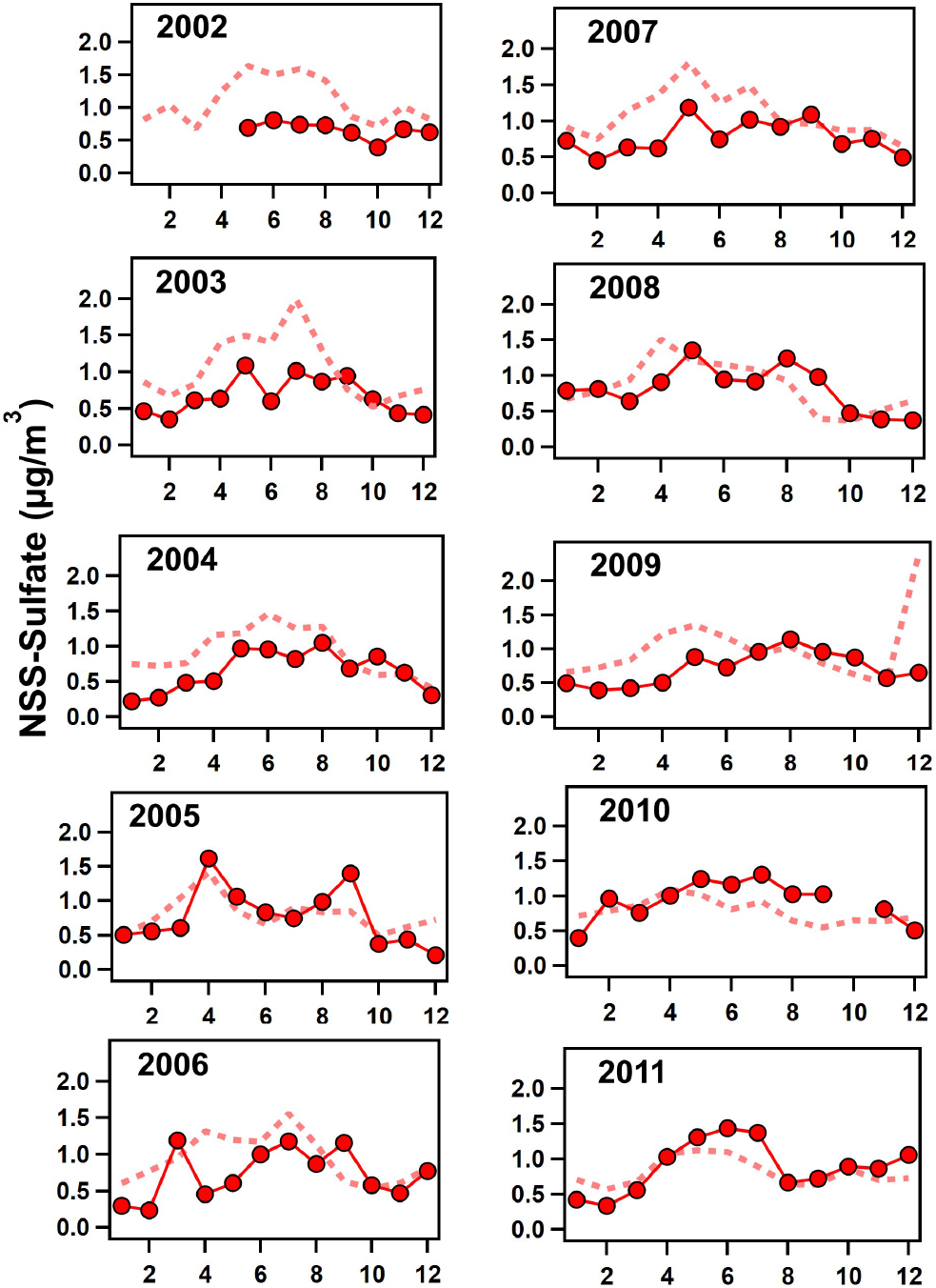
Monthly averages of non-sea-salt sulfate (nss-sulfate) mass concentrations measured in Barbados on filters (solid red line with red circles) compared to monthly averages of nss-sulfate calculated from EQUATES model simulations of combined fine- and coarse-mode sodium (Na) and sulfate using Eq. (2) (dashed red line) for 2002–2011.

**Figure 8.**
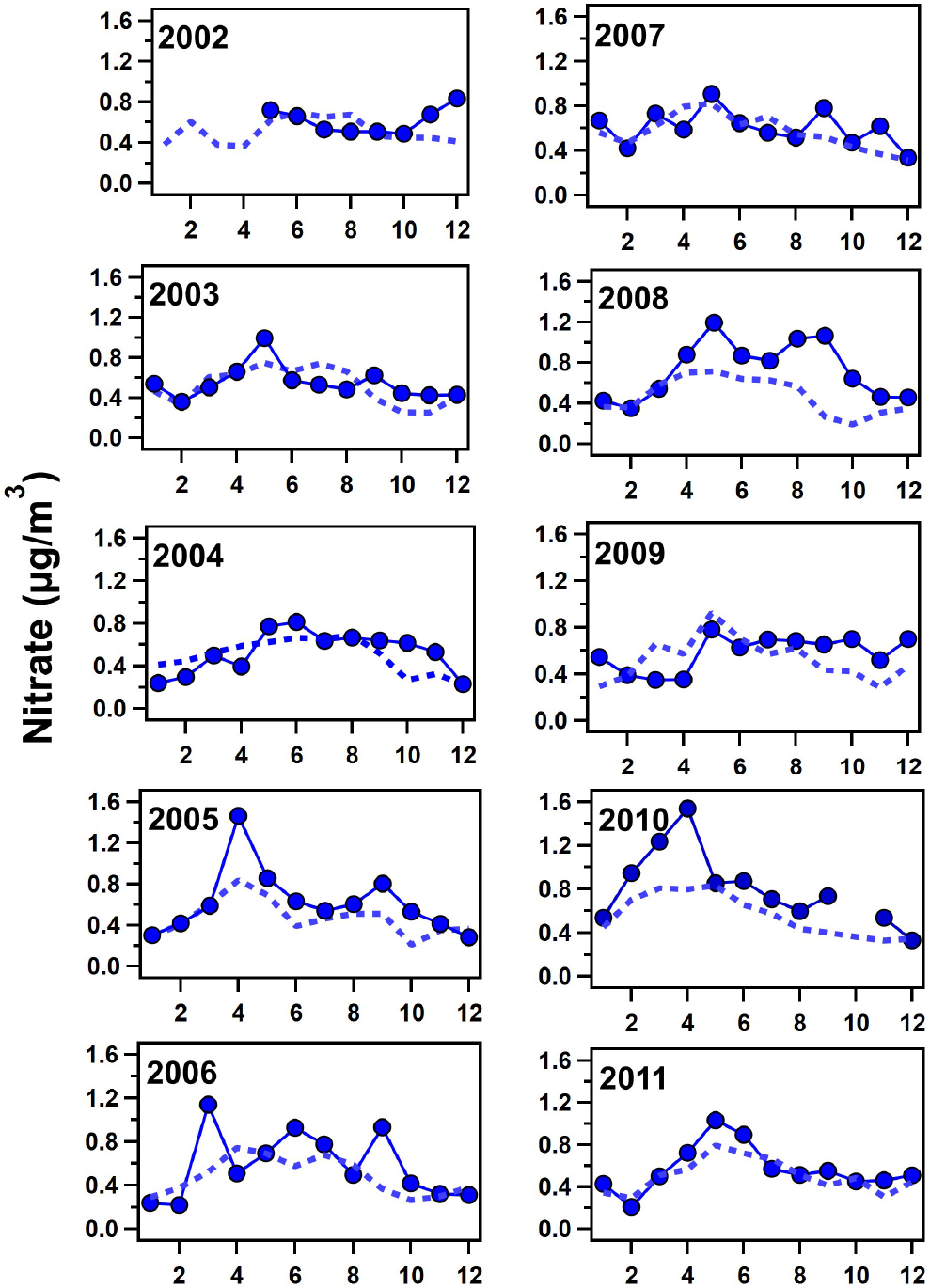
Monthly averages of nitrate mass concentrations measured in Barbados on filters (solid blue line with blue circles) compared to monthly averages of combined fine- and coarse-mode nitrate calculated from EQUATES model simulations (dashed blue line) for 2002–2011.

Monthly concentrations of simulated nss-sulfate and nitrate (for both the fine and coarse modes combined) were compared with mass concentrations measured on filters collected at Ragged Point (see Figs. 7 and 8, respectively). The model simulates similar concentrations at both Ragged Point and the area to the east of the site, implying that emissions in Barbados are minimal. EQUATES predicts nitrate concentrations in fine and coarse aerosol sizes. A greater proportion of fine nitrate is predicted in DJF (40% of the total modeled nitrate) and MAM (26% of the total modeled nitrate) during all years compared to JJA and SON (18% of the total modeled nitrate for both seasons; see Fig. S8). This seasonality is likely due to increased contributions of nitrate from fine biomass burning aerosols produced in sub-Saharan Africa north of the Equator in winter and spring. This point is highlighted in MAM of 2010 when the amount of modeled fine nitrate was elevated (35% of total modeled nitrate). nss-sulfate is predicted to be almost exclusively in fine-mode aerosols. While previous observations have shown that nss-sulfate dominates the fine mode in Barbados, some of the nss-sulfate is also present in the coarse mode likely due to heterogeneous reactions between ${\text{SO}}_{2}$ and coarse sea spray and mineral dust aerosols (Adams et al., 2005; Alexander et al., 2005; Li-Jones and Prospero, 1998). EQUATES does not seem to capture this minor yet important budget of nss-sulfate.

To assess the performance of the model compared to our observations, we calculated the normalized mean bias (NMB) and Pearson correlation coefficient (*r*) for monthly averaged concentrations of nss-sulfate and nitrate (see Table S1; Boylan and Russell, 2006). Additional calculations of the mean bias (MB) and root-mean-square error (RMSE) can also be found in Table S1 of the Supplement. The NMB for nitrate was generally within ± 20%, better than predictions of nitrate within the US (Kelly et al., 2019), except for 2005 (−24.18%), 2008 (−35.06%), and 2010 (−28.72%), when the model underpredicted measurements likely due to the elevated African smoke transport events. The model overpredicts nss-sulfate in the earlier years (2002–2007) then converges closer to our measurements at Ragged Point after 2008, as shown in Fig. 7. Because trends in sea salt (e.g., sodium from EQUATES; Fig. S1) show a constantly high bias in all years, the overprediction of nss-sulfate reflects biases in sources of sulfate (other than sea spray) or biases in the conversion of ${\text{SO}}_{2}$ to sulfate. Furthermore, the NMB for nss-sulfate also reflects the model overprediction of nss-sulfate, as high values are observed in 2002 (+81.45%), then the model begins to fall within ± 20% starting in 2008 (see Table S1). As such, trends in nss-sulfate simulated by EQUATES show a decrease over time rather than an increase post-2000 (see Fig. S9). The decrease in EQUATES simulations of nss-sulfate post-2000 compared to the increase observed in Barbados could be related to a changing bias over time. For example, recent predictions for 2019 indicate CMAQ underpredicts sulfate by about 50% in the eastern US (45% underestimation across the entire US) (Vannucci et al., 2024). Previous simulations for 2002 and 2016 indicated more modest normalized mean biases of 20% or less (Appel et al., 2021; Sarwar et al., 2011). As a result, EQUATES may simulate a stronger decline in transported US sulfate over 2002–2019 than observations indicate. This overestimation of declining sulfate in the US may mask trends (including stronger increases in sulfate) in other regions. Pearson correlation coefficients range from −0.22 to 0.88 by year for nitrate with a mean of 0.54. The poorest correlation (−0.22) occurs for 2002 when Barbados filter measurements were unavailable from January until May. For nss-sulfate, *r* ranges from 0.23 to 0.82 by year with a mean of 0.59. The greatest variation between the model and measurements is for 2009 (see Fig. 7), which was also the year that had an erroneously high model concentration of sea spray aerosols in winter. Overall, the model captures both the magnitude of and the seasonal and interannual variation in nss-sulfate and nitrate at our remote location in the tropical North Atlantic.

#### 3.2.1 Using EQUATES to determine contributions to aerosol sulfate

**Figure 9.**
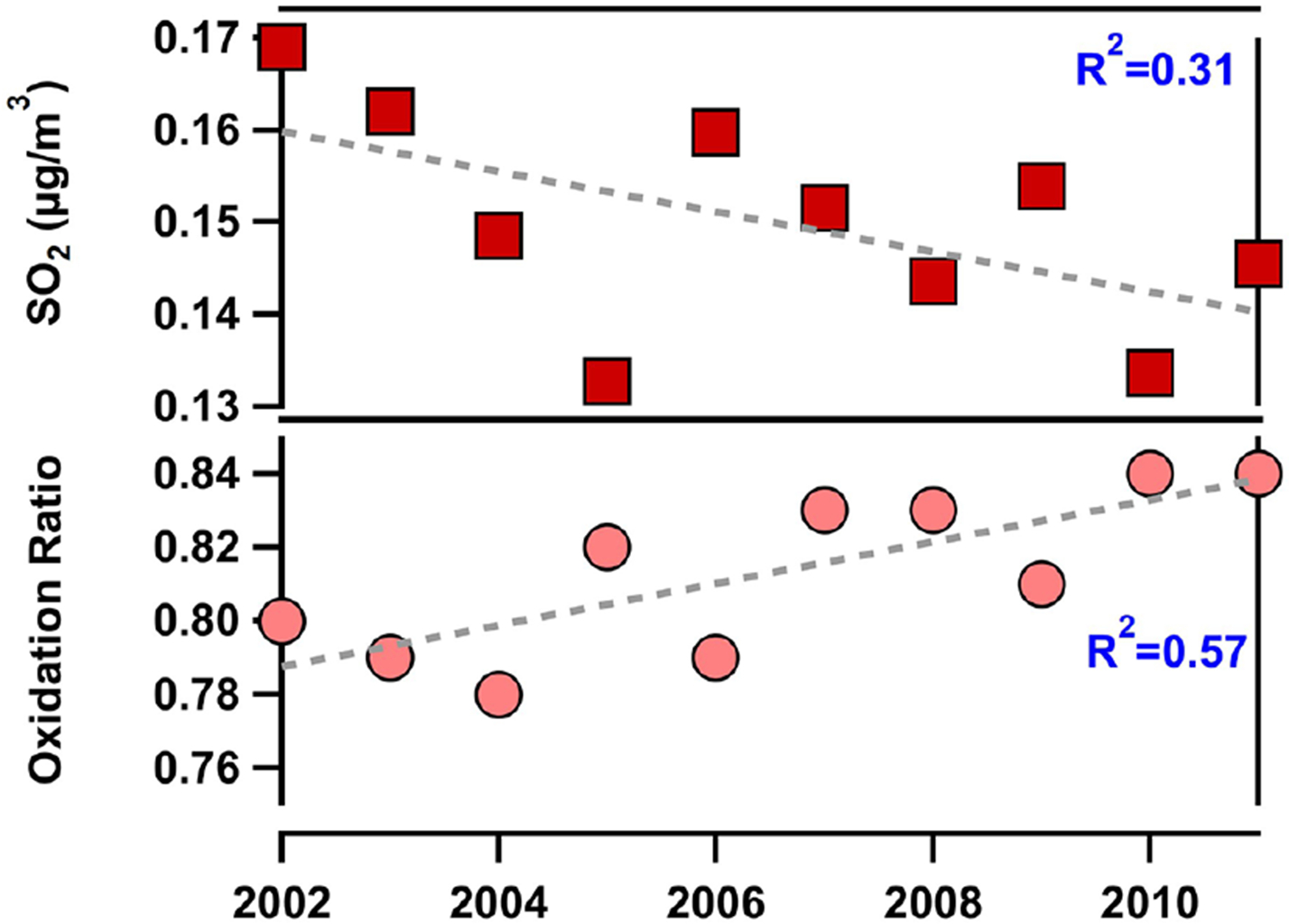
Annual trends in locally emitted sulfur dioxide (${\text{SO}}_{2}$) simulated by the EQUATES model and the calculated oxidation ratio at Ragged Point. Linear fits and corresponding correlation coefficients are also shown.

**Figure 10.**
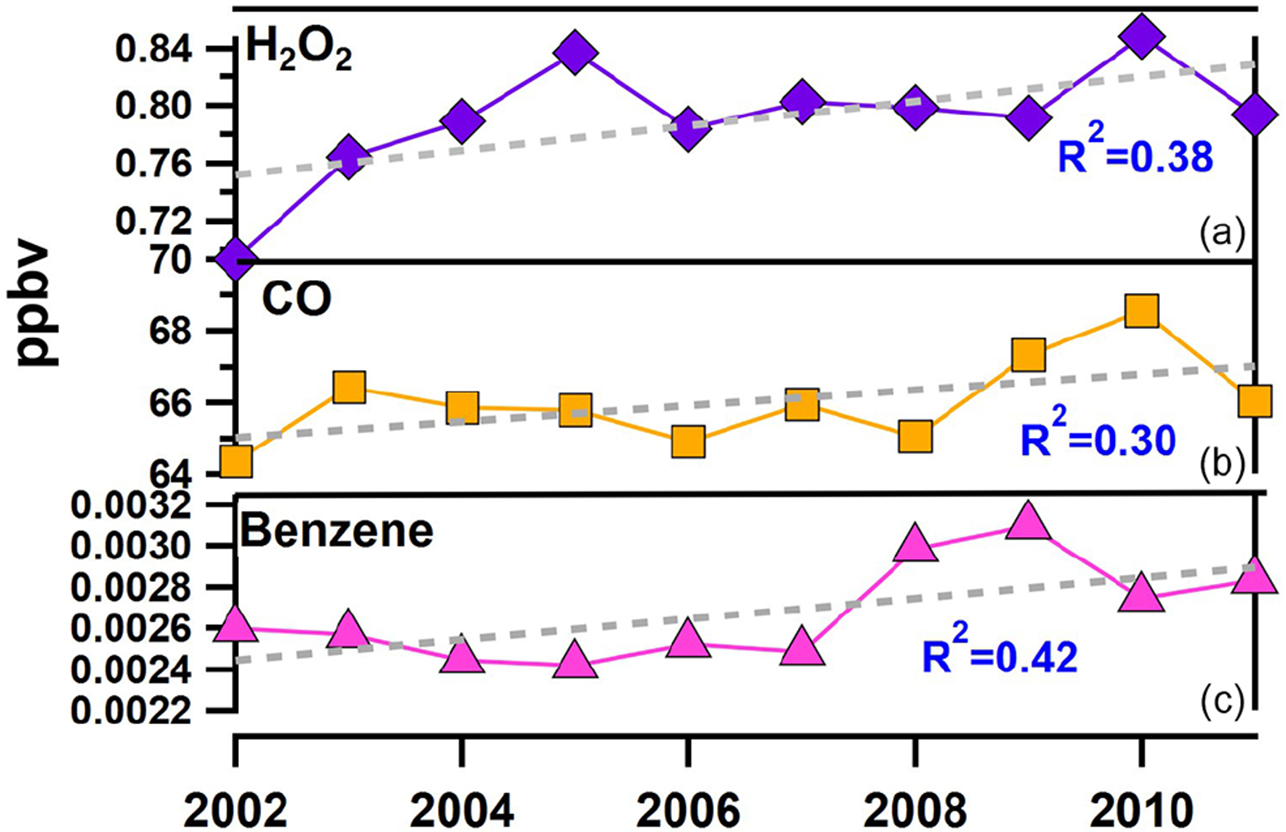
Annual trends in select gas-phase species including **(a)** hydrogen peroxide (H_2_O_2_) (purple line), **(b)** carbon monoxide (CO) (orange line), and **(c)** benzene (pink line) simulated by the EQUATES model at Ragged Point. Linear fits and corresponding correlation coefficients are also shown.

Figure 9 shows a decrease in ${\text{SO}}_{2}$ simulated by EQUATES within the modeled grid space over Ragged Point and to the east of the site. Consistent with a predicted decrease in ${\text{SO}}_{2}$ and the observed increase in nss-sulfate, the oxidation ratio of ${\text{SO}}_{2}$ increases in the region near Barbados. We note that, because the lifetime of ${\text{SO}}_{2}$ is predicted to be ~ 20 h and as much as ~ 40 h depending on the latitude of the source of ${\text{SO}}_{2}$ and the season (Green et al., 2019; Lee et al., 2011), our oxidation ratio estimates here are most relevant for local emissions, including sulfur emitted from marine phytoplankton (e.g., DMS). However, prior measurements in Bermuda suggest a longer lifetime for long-range-transported ${\text{SO}}_{2}$ compared to local oceanic emissions from DMS, which are subject to strong condensational losses to sea spray in the marine boundary layer (Keene et al., 2014). As such, long-range-transported sources from Africa, the US, and the EU were likely converted to sulfate upwind of Barbados, and ${\text{SO}}_{2}$ concentrations shown here most likely reflect oceanic sources with some contribution from long-range transport. Locally emitted hydrogen peroxide ($H_{2}O_{2}$) concentrations also increased over this period (see Fig. 10) and are likely linked to decreases in locally emitted ${\text{SO}}_{2}$, which is a major sink of $H_{2}O_{2}$ during the aqueous-phase formation of sulfate (Manktelow et al., 2007). We note that the predicted oxidation ratios are likely an overestimation because EQUATES overpredicts nss-sulfate compared to observations, and the overprediction decreases with time. As such, the increase in the oxidation ratio (Fig. 9), which is small, likely has minimal influence on the trends in nss-sulfate observed at our site.

Changes in biomass burning, anthropogenic emissions, oxidant concentrations, and meteorological parameters were also investigated using EQUATES. Fine-mode non-sea-salt potassium ($\text{nss}-K^{+}$) was used as our tracer for smoke emissions. We used EQUATES data rather than our $\text{nss}-K^{+}$ observations, which were non-existent for most years from 2002–2011. Also, our measurements included total (i.e., fine- and coarse-mode) $\text{nss}-K^{+}$, including contributions from African dust (Savoie and Prospero, 1980). EQUATES simulations of $\text{nss}-K^{+}$ were a factor of 2–3 higher than our measurements except for the dust and smoke transport event that occurred in MAM of 2010. No significant increase in simulated $\text{nss}-K^{+}$ was estimated (Fig. S10). Meteorological parameters (temperature, relative humidity (RH), wind speed, and wind direction) were constant over time, showing no shift in rainfall, winds, temperature, or RH (Fig. S10). Concentrations of the hydroxyl radical (^•^OH) did not show a significant change from 2002–2011 (Fig. S10). EQUATES concentrations of benzene (a tracer for fossil fuel combustion) and carbon monoxide (CO; a tracer for combustion from fossil fuels and biomass burning) both increased modestly from 2002–2011 (Fig. 10). Our results suggest that an increase in the oxidation efficiency of locally emitted ${\text{SO}}_{2}$ to sulfate may contribute to the increase in nss-sulfate post-2000, but the contribution is likely small compared to long-range-transported sulfate.

## 4 Discussion & conclusions

Our 21-year record (1990–2011) of nitrate and nss-sulfate aerosols shows two distinct trends over the tropical North Atlantic. Nitrate shows no significant change other than two spikes in JJA and September of 2008 and MAM of 2010. Variations in winter- and springtime dust transport explained interannual oscillations in nitrate concentrations, while increased transport of smoke from sub-Saharan Africa north of the Equator in MAM 2010 and from south of the Equator, and possibly South America, in September 2008 caused the increased levels of nitrate observed in those years. Nitrate has been shown to be enhanced in smoke by up to 5-fold over background due to high emissions of ${\text{NO}}_{x}$ that are rapidly converted to nitrate (Adon et al., 2010; Hickman et al., 2021; Perron et al., 2022; Schlosser et al., 2017). Notably, nitrate was not enhanced in JJA of 2010 even though high quantities of dust were transported to Barbados. We speculate that the lack of enhanced nitrate during the summer of 2010 is due to the lack of nitrate contribution from African smoke emissions during this season. This finding, in addition to our observations of enhanced nitrate associated with dust in DJF and MAM, suggests that the nitrate associated with African aerosol transport primarily originates from ${\text{NO}}_{x}$ emissions from African wildfires that are rapidly converted to nitrate prior to transport.

In contrast to the relatively flat trend in aerosol nitrate, nss-sulfate decreased by 30% from 1990–2000 then increased from 2000–2011, increasing to concentrations measured in the early 1990s. Reductions in nss-sulfate observed in Barbados are most likely due to decreased emissions of ${\text{SO}}_{2}$ in the US and EU due to clean air policies implemented via technologies such as flue-gas desulfurization devices installed in power plants (Aas et al., 2019; Kharol et al., 2017; Smith et al., 2011). Thus, our results highlight that regulations aimed to improve national and regional air quality also impact more distant locations such as the remote North Atlantic marine boundary layer and the Caribbean.

As shown in Fig. 6, increases in ${\text{SO}}_{2}$ in Africa, namely from anthropogenic sources, are the most probable cause for the increase in nss-sulfate levels from 2000–2011 in Barbados. Simulations from both CEDS and EQUATES reveal an increase in anthropogenic emissions of ${\text{SO}}_{2}$. Further evidence for this speculation stems from the lack of change in air mass back-trajectories before and after 2000, suggesting that emissions rather than meteorological trends are driving our observed patterns in nss-sulfate. Industrial contributions of sulfate from oil refineries, coal-fired power plants, and fertilizer plants along the north and northwestern coast of Africa were also observed in Cabo Verde (Salvador et al., 2015). Anthropogenic sources of ${\text{SO}}_{2}$ in Africa include emissions from electricity generation, diesel combustion and transportation (Assamoi and Liousse, 2010; Keita et al., 2021; Liousse et al., 2014), refineries, gas flaring, and smelting (Doumbia et al., 2019; Osuji and Avwiri, 2005). Levels of pollution in African cities are on par with Asian megacities, with the largest rate of increases in ${\text{SO}}_{2}$ observed in western Africa (Adon et al., 2016; Hopkins et al., 2009; Liousse et al., 2014; Val et al., 2013). The industrial sector in the Highveld region of South Africa also shows some of the highest increases in ${\text{SO}}_{2}$, with observed increases starting in 1980 due to an increase in the number of coal-fired power plants arising from an increased demand for electricity (Keita et al., 2021; Liousse et al., 2014; Shikwambana et al., 2020). From 1990–2011, the CEDS inventory shows the largest increase in ${\text{SO}}_{2}$ emissions in Africa starting in 2000, coincident with the increase in nss-sulfate observed at our site (see Fig. 6). The emissions inventory for Africa is also likely underestimated due to a lack of measurements (McDuffie et al., 2020). Therefore, we speculate that anthropogenic ${\text{SO}}_{2}$ emissions are likely higher than shown in the CEDS model and are driving the observed increases in nss-sulfate in Barbados.

In addition to sources of ${\text{SO}}_{2}$ within Africa, ${\text{SO}}_{2}$ emissions from other nearby countries and regions have been shown to be exported to Africa (Koch et al., 2007). In particular, ${\text{SO}}_{2}$ emissions in India have rapidly risen and overtaken China as the largest emitter of ${\text{SO}}_{2}$ (Li et al., 2017), while remote-sensing observations have highlighted that ${\text{SO}}_{2}$ emitted from oil and gas operations in the Persian Gulf have been greatly underestimated in emissions inventories (McLinden et al., 2016). While it is possible that these two regions may also contribute to the increase in nss-sulfate observed at our measurement site, we can only speculate on their importance to the remote North Atlantic marine boundary layer.

While some of the increases in nss-sulfate after 2000 could be due, in part, to marine biogenic, shipping, biomass burning, and volcanic emissions, their contributions are likely not the dominant cause of the observed trends. Marine biogenic sulfate is estimated to contribute up to 50% of nss-sulfate at Ragged Point during non-dust transport conditions (Li-Jones and Prospero, 1998; Royer et al., 2023; Savoie et al., 2002). However, our predictions of nss-sulfate and ${\text{SO}}_{2}$ concentrations with EQUATES include DMS chemistry that does not explain our observed trends. In fact, locally emitted ${\text{SO}}_{2}$, most likely from the ocean, is simulated to decrease from 2002–2011. Furthermore, while recent studies have shown links between climate change and increased DMS emissions at high latitudes, these trends have not been demonstrated at lower latitudes. Instead, DMS is predicted to decrease with increasing temperature at low latitudes due to stratification (Kloster et al., 2007) and increasing ocean acidification (Zhao et al., 2024), while a recent modeling study, factoring in changes in phytoplankton dynamics, found that DMS emissions have not appreciably changed from the preindustrial period to the present day (Wang et al., 2018). As such, DMS emissions do contribute to the sulfate burden but likely do not explain the recent increases in sulfate, which would also agree with findings in Bermuda where changes in the nss-sulfate burden were explained by anthropogenic rather than biogenic emissions (Keene et al., 2014). Shipping emissions likely do not explain our trends in nss-sulfate because Barbados is somewhat isolated from proximal shipping impacts – heavy shipping is concentrated in the Caribbean west of Barbados and along the north coast of South America (Czermanski et al., 2021). Biomass burning has declined in northern sub-Saharan Africa starting in the early 2000s (Andela et al., 2017; Andela and Van Der Werf, 2014; Zubkova et al., 2019). Furthermore, increased nss-sulfate has been observed year-round rather than just during the main burning seasons, suggesting that this source alone is likely not responsible for the major increases in nss-sulfate observed at our site. The largest natural source of ${\text{SO}}_{2}$ in Africa is volcanic emissions from Mount Nyiragongo in the Goma region of the Democratic Republic of Congo, which has been shown to impact sulfate aerosols at the Amazon Tall Tower Observatory (ATTO) in Brazil (Opio et al., 2021; Saturno et al., 2018). Volcanic emissions likely do impact nss-sulfate measured in Barbados, but there is no evidence that emissions from this source are increasing. These lines of evidence further support an anthropogenic source as the cause of the increase in nss-sulfate observed in Barbados.

In addition to increased sulfate transported from Africa, the oxidation ratio was simulated to increase from 2002–2011, which would more efficiently convert locally emitted ${\text{SO}}_{2}$ to sulfate and may represent a minor addition to the budget of nss-sulfate. Furthermore, concentrations of H_2_O_2_ increased in the post-2000 period, indicating a potential increase in the efficiency of aqueous-phase oxidation. We would expect that, even if the total burden of ${\text{SO}}_{2}$ has been reduced globally (McDuffie et al., 2020; Smith et al., 2011), ${\text{SO}}_{2}$ emitted locally is more efficiently converted to sulfate due to the greater availability of oxidants at lower latitudes (Manktelow et al., 2007). It is important to note, however, that the oxidation ratio calculated is most appropriate for accounting for changing oxidation efficiencies of ${\text{SO}}_{2}$ and sulfate formation near the site, and the oxidation ratio does not account for changes in the oxidation efficiency of already-formed sulfate aerosols that have been transported to the site. For example, ${\text{SO}}_{2}$ emitted from Africa is likely already oxidized to sulfate prior to being transported to our site.

One question that persists is why nitrate did not increase from 2000–2011 alongside the increase in nss-sulfate. One possible explanation is a combination of reduced ${\text{NO}}_{x}$ from smoke concurrent with increased dust and smoke transport that offset any changes in nitrate other than the observed spikes in 2008 and 2010. In the 2000s, biomass burning emissions declined in northern equatorial Africa due to a combination of increased precipitation in DJF associated with a shift from more frequent El Nino events in the 1990s to more frequent La Niña events in the 2000s and land use practices converting tropical savanna to cropland (Andela et al., 2017; Andela and Van Der Werf, 2014; Hickman et al., 2021; Zubkova et al., 2019). The recently updated Barbados dust record highlights that dust is being transported to the Caribbean earlier in the year and arriving more frequently in spring (Zuidema et al., 2019), which would increase the transport of biomass burning emissions and associated nitrate to the Caribbean and remote North Atlantic, which may effectively cancel out the impact of reduced smoke emissions.

The Ragged Point site in Barbados has historically been associated with research on African dust transport (Prospero et al., 2021). However, this work highlights that the site is also an excellent indicator of long-term and large-scale changes in emissions and the impact of air quality policies or the lack of them or poor compliance to them. Looking forward, building upon the existing time series of nitrate and sulfate aerosols while also expanding the measurement capabilities at Ragged Point to incorporate measurements of metals will increase our ability to apportion aerosol sources. Further, measurements of carbonaceous aerosols will provide needed insight into the impact of anthropogenic and biomass burning on sulfate and nitrate burdens over the remote North Atlantic that complements recent work performed in the South Atlantic (Zuidema et al., 2016, 2018).

## Supplementary Material

Supplement1

## Figures and Tables

**Table 1.** Seasonal trends in nitrate and nss-sulfate shown pre- and post-2000. The rate of change in μg m^−3^ yr^−1^ is shown, and the correlation coefficient is also included. Values of *R*^2^ < 0.2 are denoted as having “no trend”. DJF represents winter, MAM represents spring, JJA represents summer, and SON represents fall.

Season	Compound	Pre-2000 rate of change μg m^−3^ yr^−1^)	Post-2000 rate of change μg m^−3^ yr^−1^)
DJF	Nitrate nss-sulfate	−0.0005 (*R*^2^ = 0.05, no trend) −0.0075 (*R*^2^ = 0.05, no trend)	+0.013 (*R*^2^ = 0.07, no trend) +0.035 (*R*^2^ = 0.48)
MAM	Nitrate nss-sulfate	+0.0037 (*R*^2^ = 0.01, no trend) −0.034 (*R*^2^ = 0.64)	+0.028 (*R*^2^ = 0.08, no trend) +0.033 (*R*^2^ = 0.09, no trend)
JJA	Nitrate nss-sulfate	-0.002 (*R*^2^ = 0.02, no trend) −0.036 (*R*^2^ = 0.72)	+0.012 (*R*^2^ = 0.21) +0.028 (*R*^2^ = 0.61)
SON	Nitrate nss-sulfate	+0.0003 (*R*^2^ = 0.02, no trend) −0.023 (*R*^2^ = 0.29)	+0.013 (*R*^2^ = 0.13, no trend) +0.026 (*R*^2^ = 0.44)

## Data Availability

Measured nitrate, nss-sulfate, and sea salt concentrations can be found in the University of Miami’s repository in addition to EQUATES simulations of nitrate, nss-sulfate, sea salt, gaseous tracers (${\text{SO}}_{2}$, benzene, CO, and $H_{2}O_{2}$), and meteorological parameters (https://doi.org/10.17604/k869-9c71; Gaston et al., 2024). Dust mass concentrations from Barbados can be found at https://doi.org/10.17604/q3vf-8m31 (Zuidema, 2019). EQUATES data are available via the Remote Sensing Information Gateway (RSIG): https://www.epa.gov/hesc/remote-sensing-information-gateway (EPA, 2024).

## References

[R1] Aas W, Mortier A, Bowersox V, Cherian R, Faluvegi G, Fagerli H, Hand J, Klimont Z, Galy-Lacaux C, Lehmann CMB, Myhre CL, Myhre G, Olivié D, Sato K, Quaas J, Rao PSP, Schulz M, Shindell D, Skeie RB, Stein A, Takemura T, Tsyro S, Vet R, and Xu X: Global and regional trends of atmospheric sulfur, Sci. Rep, 9, 1–11, 10.1038/s41598-018-37304-0, 2019. 30700755 PMC6353995

[R2] Adams AM, Prospero JM, and Zhang CD: CALIPSO-derived three-dimensional structure of aerosol over the Atlantic Basin and adjacent continents, J. Climate, 25, 6862–6879, 2012.

[R3] Adams JW, Rodriguez D, and Cox RA: The uptake of ${\text{SO}}_{2}$ on Saharan dust: a flow tube study, Atmos. Chem. Phys, 5, 2679–2689, 10.5194/acp-5-2679-2005, 2005.

[R4] Adams PJ, Seinfeld JH, and Koch DM: Global concentrations of tropospheric sulfate, nitrate, and ammonium aerosol simulated in a general circulation model, J. Geophys. Res, 104, 13791–13823, 10.1029/1999JD900083, 1999.

[R5] Adebiyi AA and Zuidema P: The role of the southern African easterly jet in modifying the southeast Atlantic aerosol and cloud environments, Q. J. Roy, 142, 1574–1589, 2016.

[R6] Adon M, Galy-Lacaux C, Yoboué V, Delon C, Lacaux JP, Castera P, Gardrat E, Pienaar J, Al Ourabi H, Laouali D, Diop B, Sigha-Nkamdjou L, Akpo A, Tathy JP, Lavenu F, and Mougin E: Long term measurements of sulfur dioxide, nitrogen dioxide, ammonia, nitric acid and ozone in Africa using passive samplers, Atmos. Chem. Phys, 10, 7467–7487, 10.5194/acp-10-7467-2010, 2010.

[R7] Adon M, Yobou V, Galy-Lacaux C, Liousse C, Diop B, Hadji E, Doumbia T, Gardrat E, Ndiaye SA, and Jarnot C: Measurements of NO_2_, ${\text{SO}}_{2}$, NH_3_, HNO_3_ and O_3_ in West African urban environments, Atmos. Environ, 135, 31–40, 10.1016/j.atmosenv.2016.03.050, 2016.

[R8] Alexander B, Park RJ, Jacob DJ, Li QB, Yantosca RM, Savarino J, Lee CCW, and Thiemens MH: Sulfate formation in sea-salt aerosols: Constraints from oxygen isotopes, J. Geophys. Res, 110, 1–12, 10.1029/2004JD005659, 2005.

[R9] Andela N and Van Der Werf GR: Recent trends in African fires driven by cropland expansion and El Niño to La Niña transition, Nat. Clim. Change, 4, 791–795, 10.1038/NCLIMATE2313, 2014.

[R10] Andela N, Morton DC, Giglio L, Chen Y, Van Der Werf GR, Kasibhatla PS, DeFries RS, Collatz GJ, Hantson S, Kloster S, Bachelet D, Forrest M, Lasslop G, Li F, Mangeon S, Melton JR, Yue C, and Randerson JT: A human-driven decline in global burned area, Science, 356, 1356–1362, 10.1126/science.aal4108, 2017. 28663495 PMC6047075

[R11] Andreae MO, Ferek RJ, Bermond F, Byrd KP, Engstrom RT, Hardin S, Houmere PD, LeMarrec F, and Raemdonck H: Dimethyl sulfide in the marine atmosphere, J. Geophys. Res, 90, 12891–12900, 1985.

[R12] Andreae MO: Soot carbon and excess fine potassium: Long-range transport of combustion-derived aerosols, Science, 220, 1148–1151, 10.1126/SCIENCE.220.4602.1148, 1983. 17818494

[R13] Andreae MO: Emission of trace gases and aerosols from biomass burning – an updated assessment, Atmos. Chem. Phys, 19, 8523–8546, 10.5194/acp-19-8523-2019, 2019.

[R14] Andreae MO and Merlet P: Emission of trace gases and aerosols from biomass burning, Global Biogeochem. Cy, 15, 955–966, 2001.

[R15] Appel BR, Kothny EL, Hoffer EM, Hidy GM, and Wesolowski JJ: Sulfate and nitrate data from California Aerosol Characterization Experiment (ACHEX), Environ. Sci. Technol, 12, 418–425, 1978.

[R16] Appel KW, Bash JO, Fahey KM, Foley KM, Gilliam RC, Hogrefe C, Hutzell WT, Kang D, Mathur R, Murphy BN, Napelenok SL, Nolte CG, Pleim JE, Pouliot GA, Pye HOT, Ran L, Roselle SJ, Sarwar G, Schwede DB, Sidi FI, Spero TL, and Wong DC: The Community Multiscale Air Quality (CMAQ) model versions 5.3 and 5.3.1: system updates and evaluation, Geosci. Model Dev, 14, 2867–2897, 10.5194/gmd-14-2867-2021, 2021. 34676058 PMC8525427

[R17] Assamoi EM and Liousse C: A new inventory for two-wheel vehicle emissions in West Africa for 2002, Atmos. Environ, 44, 3985–3996, 10.1016/J.ATMOSENV.2010.06.048, 2010.

[R18] Barkley AE, Prospero JM, Mahowald N, Hamilton DS, Popendorf KJ, Oehlert AM, Pourmand A, Gatineau A, Panechou-Pulcherie K, Blackwelder P, and Gaston CJ: African biomass burning is a substantial source of phosphorus deposition to the Amazon, Tropical Atlantic Ocean, and Southern Ocean, P. Natl. Acad. Sci. USA, 116, 16216–16221, 10.1073/pnas.1906091116, 2019. PMC6697889 31358622

[R19] Barkley AE, Olson NE, Prospero JM, Gatineau A, Panechou K, Maynard NG, Blackwelder P, China S, Ault AP, and Gaston CJ: Atmospheric transport of North African dust-bearing supermicron freshwater diatoms to South America: Implications for iron transport to the equatorial North Atlantic Ocean, Geophys. Res. Lett, 48, e2020GL090476, 10.1029/2020GL090476, 2021.

[R20] Barnes I, Hjorth J, and Mihalopoulos N: Dimethyl sulfide and dimethyl sulfoxide and their oxidation in the atmosphere, Chem. Rev, 106, 940–975, 2006. 16522014 10.1021/cr020529+

[R21] Benish SE, Bash JO, Foley KM, Appel KW, Hogrefe C, Gilliam R, and Pouliot G: Long-term regional trends of nitrogen and sulfur deposition in the United States from 2002 to 2017, Atmos. Chem. Phys, 22, 12749–12767, 10.5194/acp-22-12749-2022, 2022. PMC11864274 40012769

[R22] Boylan JW and Russell AG: PM and light extinction model performance metrics, goals, and criteria for three-dimensional air quality models, Atmos. Environ, 40, 4946–4959, 10.1016/j.atmosenv.2005.09.087, 2006.

[R23] Carlson TN and Prospero JM: The large-scale movement of Saharan air outbreaks over the Northern Equatorial Atlantic, J. Appl. Meteorol, 11, 283–297, 1972.

[R24] Carslaw KS, Lee LA, Reddington CL, Pringle KJ, Rap A, Forster PM, Mann GW, Spracklen DV, Woodhouse MT, Regayre LA, and Pierce JR: Large contribution of natural aerosols to uncertainty in indirect forcing, Nature, 503, 67–71, 10.1038/nature12674, 2013. 24201280

[R25] Charlson RJ, Schwartz SE, Hales JM, Cess RD, Coakley JA, Hansen JE, and Hofmann DJ: Climate forcing by anthropogenic aerosols, Science, 255, 423–430, 10.1126/SCIENCE.255.5043.423, 1992. 17842894

[R26] Chiapello I, Moulin C, and Prospero JM: Understanding the long-term variability of African dust transport across the Atlantic as recorded in both Barbados surface concentrations and large-scale Total Ozone Mapping Spectrometer (TOMS) optical thickness, J. Geophys. Res, 110, 1–9, 10.1029/2004JD005132, 2005.

[R27] Czermański E, Cirella GT, Notteboom T, Oniszczuk-Jastrzabek A, and Pawłowska B: An energy consumption approach to estimate air emission reductions in container shipping, Energies, 14, 278, 10.3390/EN14020278, 2021.

[R28] Doherty OM, Riemer N, and Hameed S: Saharan mineral dust transport into the Caribbean: Observed atmospheric controls and trends, J. Geophys. Res, 113, D07211, 10.1029/2007JD009171, 2008.

[R29] Doherty OM, Riemer N, and Hameed S: Control of Saharan mineral dust transport to Barbados in winter by the Intertropical Convergence Zone over West Africa, J. Geophys. Res, 117, 19117, 10.1029/2012JD017767, 2012.

[R30] Doumbia EHT, Liousse C, Keita S, Granier L, Granier C, Elvidge CD, Elguindi N, and Law K: Flaring emissions in Africa: Distribution, evolution and comparison with current inventories, Atmos. Environ, 199, 423–434, 10.1016/J.ATMOSENV.2018.11.006, 2019.

[R31] Draxler RR and Rolph GD: HYSPLIT (HYbrid Single-Particle Lagrangian Integrated Trajectory) Model access via NOAA ARL READY Website (http://ready.arl.noaa.gov/HYSPLIT.php, last access: 21 May 2024), NOAA Air Resources Laboratory, Silver Spring, MD, 2011.

[R32] EPA: Our Nation’s Air, Trends through 2022, https://gispub.epa.gov/air/trendsreport/2023/ (last access: 21 May 2024), 2023a.

[R33] EPA: High-End Scientific Computing, Remote Sensing Information Gateway, https://www.epa.gov/hesc/remote-sensing-information-gateway (last access:10 July 2024), 2024.

[R34] Foley KM, Pouliot GA, Eyth A, Aldridge MF, Allen C, Appel KW, Bash JO, Beardsley M, Beidler J, Choi D, Farkas C, Gilliam RC, Godfrey J, Henderson BH, Hogrefe C, Koplitz SN, Mason R, Mathur R, Misenis C, Possiel N, Pye HOT, Reynolds L, Roark M, Roberts S, Schwede DB, Seltzer KM, Sonntag D, Talgo K, Toro C, Vukovich J, Xing J, and Adams E: 2002–2017 anthropogenic emissions data for air quality modeling over the United States, Data in Brief, 47, 109022, 10.1016/J.DIB.2023.109022, 2023. 36942100 PMC10023994

[R35] Galloway JN, Townsend AR, Erisman JW, Bekunda M, Cai Z, Freney JR, Martinelli LA, Seitzinger SP, and Sutton MA: Transformation of the nitrogen cycle: Recent trends, questions, and potential solutions, Science, 320, 889–892, 10.1126/SCIENCE.1136674, 2008. 18487183

[R36] Gaston CJ, Prospero JM, Foley K, Pye HOT, Custals L, Blades E, Sealy P and Christie JA: Daily nitrate, sulfate, and sea salt aerosol mass concentrations measured at Ragged Point, Barbados from 1990–2011 and aerosol and meteorological model predictions from EQUATES, University of Miami Libraries [data set], 10.17604/k869-9c71, 2024.

[R37] Giglio L, Randerson JT, and van der Werf GR: Analysis of daily, monthly, and annual burned area using the fourth-generation global fire emissions database (GFED4), J. Geophys. Res, 118, 317–328, 2013.

[R38] Giglio L, Csiszar I, and Justice CO: Global distribution and seasonality of active fires as observed with the Terra and Aqua Moderate Resolution Imaging Spectrora-diometer (MODIS) sensors, J. Geophys. Res, 111, G02016, 10.1029/2005JG000142, 2006.

[R39] Goudie AS and Middleton NJ: Saharan dust storms: nature and consequences, Earth-Sci. Rev, 56, 179–204, 10.1016/S0012-8252(01)00067-8, 2001.

[R40] Green JR, Fiddler MN, Holloway JS, Fibiger DL, McDuffie EE, Campuzano-Jost P, Schroder JC, Jimenez JL, Weinheimer AJ, Aquino J, Montzka DD, Hall SR, Ullmann K, Shah V, Jaegld L, Thornton JA, Bililign S, and Brown SS: Rates of wintertime atmospheric ${\text{SO}}_{2}$ oxidation based on aircraft observations during clear-sky conditions over the Eastern United States, J. Geophys. Res, 124, 6630–6649, 10.1029/2018JD030086, 2019.

[R41] Gutleben M, Groß S, Heske C, and Wirth M: Wintertime Saharan dust transport towards the Caribbean: an airborne lidar case study during EUREC4A, Atmos. Chem. Phys, 22, 7319–7330, 10.5194/acp-22-7319-2022, 2022.

[R42] Hand JL, Schichtel BA, Malm WC, and Pitchford ML: Particulate sulfate ion concentration and ${\text{SO}}_{2}$ emission trends in the United States from the early 1990s through 2010, Atmos. Chem. Phys, 12, 10353–10365, 10.5194/acp-12-10353-2012, 2012.

[R43] Hand JL, Prenni AJ, and Schichtel BA: Trends in seasonal mean speciated aerosol composition in remote areas of the United States From 2000 through 2021, J. Geophys. Res, 129, e2023JD039902. 10.1029/2023JD039902, 2024. PMC11608814 39624178

[R44] Hickman JE, Andela N, Tsigaridis K, Galy-Lacaux C, Ossohou M, and Bauer SE: Reductions in NO_2_ burden over north equatorial Africa from decline in biomass burning in spite of growing fossil fuel use, 2005 to 2017, P. Natl. Acad. Sci. USA, 118, e2002579118, 10.1073/pnas.2002579118, 2021. PMC7896302 33558224

[R45] Hopkins JR, Evans MJ, Lee JD, Lewis AC, Marsham JH, McQuaid JB, Parker DJ, Stewart DJ, Reeves CE, and Purvis RM: Direct estimates of emissions from the megacity of Lagos, Atmos. Chem. Phys, 9, 8471–8477, 10.5194/acp-9-8471-2009, 2009.

[R46] Jickells TD, Buitenhuis E, Altieri K, Baker AR, Capone D, Duce RA, Dentener F, Fennel K, Kanakidou M, LaRoche J, Lee K, Liss P, Middelburg JJ, Moore JK, Okin G, Oschlies A, Sarin M, Seitzinger S, Sharples J, Singh A, Suntharalingam P, Uematsu M, and Zamora LM: A reevaluation of the magnitude and impacts of anthropogenic atmospheric nitrogen inputs on the ocean, Global Biogeochem. Cycles, 31, 289–305, 10.1002/2016GB005586, 2017.

[R47] Keene WC, Pszenny AAP, Galloway JN, and Hawley ME: Sea-salt corrections and interpretation of constituent ratios in marine precipitation, J. Geophys. Res, 91, 6647, 10.1029/JD091ID06P06647, 1986.

[R48] Keene WC, Moody JL, Galloway JN, Prospero JM, Cooper OR, Eckhardt S, and Maben JR: Long-term trends in aerosol and precipitation composition over the western North Atlantic Ocean at Bermuda, Atmos. Chem. Phys, 14, 8119–8135, 10.5194/acp-14-8119-2014, 2014.

[R49] Keita S, Liousse C, Assamoi E-M, Doumbia T, N’Datchoh ET, Gnamien S, Elguindi N, Granier C, and Yoboué V: African anthropogenic emissions inventory for gases and particles from 1990 to 2015, Earth Syst. Sci. Data, 13, 3691–3705, 10.5194/essd-13-3691-2021, 2021.

[R50] Kelly JT, Koplitz SN, Baker KR, Holder AL, Pye HOT, Murphy BN, Bash JO, Henderson BH, Possiel NC, Simon H, Eyth AM, Jang C, Phillips S, and Timin B: Assessing PM_2.5_ model performance for the conterminous U.S. with comparison to model performance statistics from 2007–2015, Atmos. Environ, 214, 116872, 10.1016/j.atmosenv.2019.116872, 2019. PMC6859642 31741655

[R51] Kganyago M and Shikwambana L: Assessing spatio-temporal variability of wildfires and their impact on Sub-Saharan ecosystems and air quality using multisource remotely sensed data and trend analysis, Sustainability, 11, 6811, 10.3390/su11236811, 2019.

[R52] Kharol SK, McLinden CA, Sioris CE, Shephard MW, Fioletov V, van Donkelaar A, Philip S, and Martin RV: OMI satellite observations of decadal changes in ground-level sulfur dioxide over North America, Atmos. Chem. Phys, 17, 5921–5929, 10.5194/acp-17-5921-2017, 2017.

[R53] Kloster S, Six KD, Feichter J, Maier-Reimer E, Roeckner E, Wetzel P, Stier P, and Esch M: Response of dimethylsulfide (DMS) in the ocean and atmosphere to global warming, J. Geophys. Res, 112, G03005, 10.1029/2006JG000224, 2007.

[R54] Koch D, Bond TC, Streets D, Unger N, and van der Werf GR: Global impacts of aerosols from particular source regions and sectors, J. Geophys. Res, 112, 2205, 10.1029/2005JD007024, 2007.

[R55] Kramer SJ, Kirtman BP, Zuidema P, and Ngan F : Subseasonal variability of elevated dust concentrations over South Florida, J. Geophys. Res, 125, e2019JD031874, 10.1029/2019JD031874, 2020.

[R56] Lee C, Martin RV, Van Donkelaar A, Lee H, Dickerson RR, Hains JC, Krotkov N, Richter A, Vinnikov K, and Schwab JJ: ${\text{SO}}_{2}$ emissions and lifetimes: Estimates from inverse modeling using in situ and global, space-based (SCIAMACHY and OMI) observations, J. Geophys. Res, 116, D06304, 10.1029/2010JD014758, 2011.

[R57] Lelieveld J and Heintzenberg J: Sulfate cooling effect on climate through in-cloud processing of anthropogenic ${\text{SO}}_{2}$, Science, 258, 117–120, 1992. 17835896 10.1126/science.258.5079.117

[R58] Lelieveld J, Berresheim H, Borrmann S, Crutzen PJ, Dentener FJ, Fischer H, Feichter J, Flatau PJ, Heland J, Holzinger R, Korrmann R, Lawrence MG, Levin Z, Markowicz KM, Mihalopoulos N, Minikin A, Ramanathan V, De Reus M, Roelofs GJ, Scheeren HA, Sciare J, Schlager H, Schultz M, Siegmund P, Steil B, Stephanou EG, Stier P, Traub M, Warneke C, Williams J, and Ziereis G : Global air pollution crossroads over the Mediterranean, Science, 298, 794–799, 2002. 12399583 10.1126/science.1075457

[R59] Lelieveld J, Hoor P, Jöckel P, Pozzer A, Hadjinicolaou P, Cammas J-P, and Beirle S: Severe ozone air pollution in the Persian Gulf region, Atmos. Chem. Phys, 9, 1393–1406, 10.5194/acp-9-1393-2009, 2009.

[R60] Li C, McLinden C, Fioletov V, Krotkov N, Carn S, Joiner J, Streets D, He H, Ren X, Li Z, and Dickerson RR: India is overtaking China as the world’s largest emitter of anthropogenic sulfur dioxide, Sci. Rep, 7, 1–7, 10.1038/s41598-017-14639-8, 2017. 29123116 PMC5680191

[R61] Li-Jones X and Prospero JM: Variations in the size distribution of non-sea-salt sulfate aerosol in the marine boundary layer at Barbados: Impact of African dust, J. Geophys. Res, 103, 16073–16084, 1998.

[R62] Liousse C, Assamoi E, Criqui P, Granier C, and Rosset R: Explosive growth in African combustion emissions from 2005 to 2030, Environ. Res. Lett, 9, 035003, 10.1088/1748-9326/9/3/035003, 2014.

[R63] Mahowald N, Scanza R, Brahney J, Goodale CL, Hess PG, Moore JK, and Neff J: Aerosol deposition impacts on land and ocean carbon cycles, Curr. Clim. Change Rep, 3, 16–31, 2017.

[R64] Manktelow PT, Mann GW, Carslaw KS, Spracklen DV, and Chipperfield MP: Regional and global trends in sulfate aerosol since the 1980s, Geophys. Res. Lett, 34, 14803, 10.1029/2006GL028668, 2007.

[R65] Mathur R, Xing J, Gilliam R, Sarwar G, Hogrefe C, Pleim J, Pouliot G, Roselle S, Spero TL, Wong DC, and Young J: Extending the Community Multiscale Air Quality (CMAQ) modeling system to hemispheric scales: overview of process considerations and initial applications, Atmos. Chem. Phys, 17, 12449–12474, 10.5194/acp-17-12449-2017, 2017. 29681922 PMC5907506

[R66] McDuffie EE, Smith SJ, O’Rourke P, Tibrewal K, Venkataraman C, Marais EA, Zheng B, Crippa M, Brauer M, and Martin RV: A global anthropogenic emission inventory of atmospheric pollutants from sector- and fuel-specific sources (1970–2017): an application of the Community Emissions Data System (CEDS), Earth Syst. Sci. Data, 12, 3413–3442, 10.5194/essd-12-3413-2020, 2020.

[R67] McLinden CA, Fioletov V, Shephard MW, Krotkov N, Li C, Martin RV, Moran MD, and Joiner J: Space-based detection of missing sulfur dioxide sources of global air pollution, Nat. Geosci, 9, 496–500, 10.1038/NGEO2724, 2016.

[R68] Murphy DM, Cziczo DJ, Froyd KD, Hudson PK, Matthew BM, Middlebrook AM, Peltier RE, Sullivan A, Thomson DS, and Weber RJ: Single-particle mass spectrometry of tropospheric aerosol particles, J. Geophys. Res, 111, D23S32, 10.1029/2006JD007340, 2006.

[R69] Nowell HK, Holmes CD, Robertson K, Teske C, and Hiers JK: A new picture of fire extent, variability, and drought interaction in prescribed fire landscapes: Insights from Florida Government Records, Geophys. Res. Lett, 45, 7874–7884, 10.1029/2018GL078679, 2018. 31031448 PMC6474124

[R70] Opio R, Mugume I, and Nakatumba-Nabende J: Understanding the trend of NO_2_, ${\text{SO}}_{2}$ and CO over East Africa from 2005 to 2020, Atmosphere, 12, 1283, 10.3390/ATMOS12101283, 2021.

[R71] Osuji LC and Avwiri GO: Flared gases and other pollutants associated with air quality in industrial areas of Nigeria: An overview, Chem. Biodiversity, 2, 1277–1289, 10.1002/CBDV.200590099, 2005. 17191928

[R72] Perron MMG, Meyerink S, Corkill M, Strzelec M, Proemse BC, Gault-Ringold M, Sanz Rodriguez E, Chase Z, and Bowie AR: Trace elements and nutrients in wildfire plumes to the southeast of Australia, Atmos. Res, 270, 106084, 10.1016/J.ATMOSRES.2022.106084, 2022.

[R73] Prospero JM: Long-term measurements of the transport of African mineral dust to the southeastern United States: Implications for regional air quality, J. Geophys. Res, 104, 15917–15927, 1999.

[R74] Prospero JM and Mayol-Bracero OL: Understanding the transport and impact of African dust on the Caribbean Basin, B. Am. Meteor. Soc, 94, 1329–1337, 2013.

[R75] Prospero JM, Glaccum RA, and Nees RT: Atmospheric transport of soil dust from Africa to South America, Nature, 289, 570–572, 1981.

[R76] Prospero JM, Olmez I, and Ames M: Al and Fe in PM_2.5_ and PM_10_ suspended particles in South-Central Florida: The impact of the long range transport of African mineral dust, Wat. Air Soil Poll, 125, 291–317, 2001.

[R77] Prospero JM, Collard F-X, Molinie J, and Jeannot A: Characterizing the annual cycle of African dust transport to the Caribbean Basin and South America and its impact on the environment and air quality, Global Biogeochem. Cy, 29, 757–773, 10.1111/1462-2920.13280, 2014.

[R78] Prospero JM, Delany AC, Delany AC, and Carlson TN: The discovery of African dust transport to the western hemisphere and the Saharan Air Layer: A history, B. Am. Meteor. Soc, 102, E1239–E1260, 10.1175/BAMS-D-19-0309.1, 2021.

[R79] Quinn PK, Thompson EJ, Coffman DJ, Baidar S, Bariteau L, Bates TS, Bigorre S, Brewer A, de Boer G, de Szoeke SP, Drushka K, Foltz GR, Intrieri J, Iyer S, Fairall CW, Gaston CJ, Jansen F, Johnson JE, Krüger OO, Marchbanks RD, Moran KP, Noone D, Pezoa S, Pincus R, Plueddemann AJ, Pöhlker ML, Pöschl U, Quinones Melendez E, Royer HM, Szczodrak M, Thomson J, Upchurch LM, Zhang C, Zhang D, and Zuidema P: Measurements from the RV *Ronald H. Brown* and related platforms as part of the Atlantic Tradewind Ocean-Atmosphere Mesoscale Interaction Campaign (ATOMIC), Earth Syst. Sci. Data, 13,1759–1790, 10.5194/essd-13-1759-2021, 2021.

[R80] Quinn PK, Bates TS, Coffman DJ, Upchurch LM, Johnson JE, Brewer A, Baidar S, McCoy IL, and Zuidema P: Wintertime observations of tropical northwest Atlantic aerosol properties during ATOMIC: Varying mixtures of dust and biomass burning, J. Geophys. Res, 127, e2021JD036253, 10.1029/2021JD036253, 2022.

[R81] Rafaj P, Amann M, Siri J, and Wuester H: Changes in European greenhouse gas and air pollutant emissions 1960–2010: decomposition of determining factors (Uncertainties in Greenhouse Gas Inventories), edited by: Ometto J, Bun R, Jonas M, Nahorski Z, Springer, Cham, 27–54, 10.1007/978-3-319-15901-0_3, 2015.

[R82] Rickly PS, Guo H, Campuzano-Jost P, Jimenez JL, Wolfe GM, Bennett R, Bourgeois I, Crounse JD, Dibb JE, DiGangi JP, Diskin GS, Dollner M, Gargulinski EM, Hall SR, Halliday HS, Hanisco TF, Hannun RA, Liao J, Moore R, Nault BA, Nowak JB, Peischl J, Robin-son CE, Ryerson T, Sanchez KJ, Schöberl M, Soja AJ, St. Clair JM, Thornhill KL, Ullmann K, Wennberg PO, Weinzierl B, Wiggins EB, Winstead EL, and Rollins AW: Emission factors and evolution of ${\text{SO}}_{2}$ measured from biomass burning in wildfires and agricultural fires, Atmos. Chem. Phys, 22, 15603–15620, 10.5194/acp-22-15603-2022, 2022.

[R83] Roberts G, Wooster MJ, and Lagoudakis E: Annual and diurnal african biomass burning temporal dynamics, Biogeosciences, 6, 849–866, 10.5194/bg-6-849-2009, 2009.

[R84] Rolph G, Stein A, and Stunder B: Real-time Environmental Applications and Display sYstem: READY, Environ. Model. Softw, 95, 210–228, 2017.

[R85] Royer HM, Pöhlker ML, Krüger O, Blades E, Sealy P, Lata NN, Cheng Z, China S, Ault AP, Quinn PK, Zuidema P, Pöhlker C, Pöschl U, Andreae M, and Gaston CJ: African smoke particles act as cloud condensation nuclei in the wintertime tropical North Atlantic boundary layer over Barbados, Atmos. Chem. Phys, 23, 981–998, 10.5194/acp-23-981-2023, 2023.

[R86] Salvador P, Almeida SM, Cardoso J, Almeida-Silva M, Nunes T, Cerqueira M, Alves C, Reis MA, Chaves PC, Artínano B, and Pio C: Composition and origin of PM_10_ in Cape Verde: Characterization of long-range transport episodes, Atmos. Environ, 127, 326–339, 2015.

[R87] Sarwar G, Fahey KM, Napelenok SL, Roselle SJ, and Mathur R: Examining the impact of CMAQ model updates on aerosol sulfate predictions, in: The 10th Annual CMAS Models-3 User’s Conference (p. vol 775), Chapel Hill, NC, https://cmascenter.org/conference/2011/slides/sarwar_examining_impact_2011.pdf (last access: 10 July 2024), 2011.

[R88] Sarwar G, Hogrefe C, Henderson BH, Foley K, Mathur R, Murphy B, and Ahmed S: Characterizing variations in ambient PM_2.5_ concentrations at the U.S. Embassy in Dhaka, Bangladesh using observations and the CMAQ modeling system, Atmos. Environ, 296, 119587, 10.1016/J.ATMOSENV.2023.119587, 2023. PMC10581604 37854171

[R89] Saturno J, Ditas F, Penning de Vries M, Holanda BA, Pöhlker ML, Carbone S, Walter D, Bobrowski N, Brito J, Chi X, Gutmann A, Hrabe de Angelis I, Machado LAT, Moran-Zuloaga D, Rödiger J, Schneider J, Schulz C, Wang Q, Wendisch M, Artaxo P, Wagner T, Poschl U, Andreae MO, and Pöhlker C: African volcanic emissions influencing atmospheric aerosols over the Amazon rain forest, Atmos. Chem. Phys, 18, 10391–10405, 10.5194/acp-18-10391-2018, 2018.

[R90] Savoie DL and Prospero JM: Water-soluble potassium, calcium, and magnesium in the aerosols over the tropical North Atlantic, J. Geophys. Res, 85, 385–392, 10.1029/JC085IC01P00385, 1980.

[R91] Savoie DL and Prospero JM: Particle size distribution of nitrate and sulfate in the marine atmosphere, Geophys. Res. Lett, 9, 1207–1210, 1982.

[R92] Savoie DL, Arimoto R, Keene WC, Prospero JM, Duce RA, and Galloway JN: Marine biogenic and anthropogenic contributions to non-sea-salt sulfate in the marine boundary layer over the North Atlantic Ocean, J. Geophys. Res, 107, 4356, 10.1029/2001JD000970, 2002.

[R93] Scheuvens D, Schutz L, Kandler K, Ebert M, and Weinbruch S : Bulk composition of northern African dust and its source sediments – A compilation, Earth-Sci. Rev, 116, 170–194, 2013.

[R94] Schlosser JS, Braun RA, Bradley T, Dadashazar H, Mac-Donald AB, Aldhaif AA, Aghdam MA, Mardi AH, Xian P, and Sorooshian A: Analysis of aerosol composition data for western United States wildfires between 2005 and 2015: Dust emissions, chloride depletion, and most enhanced aerosol constituents, J. Geophys. Res, 122, 8951–8966, 10.1002/2017JD026547, 2017. PMC5611831 28955601

[R95] Shah V, Jaeglé L, Thornton JA, Lopez-Hilfiker FD, Lee BH, Schroder JC, Campuzano-Jost P, Jimenez JL, Guo H, Sullivan AP, Weber RJ, Green JR, Fiddler MN, Bililign S, Campos TL, Stell M, Weinheimer AJ, Montzka DD, and Brown SS: Chemical feedbacks weaken the wintertime response of particulate sulfate and nitrate to emissions reductions over the eastern United States, P. Natl. Acad. Sci. USA, 115, 8110–8115, 10.1073/PNAS.1803295115, 2018. PMC6094106 30037992

[R96] Shikwambana L, Mhangara P, and Mbatha N: Trend analysis and first time observations of sulphur dioxide and nitrogen dioxide in South Africa using TROPOMI/Sentinel-5 P data, Int. J. Appl. Earth Obs. Geoinf, 91, 102130, 10.1016/J.JAG.2020.102130, 2020.

[R97] Smith SJ, van Aardenne J, Klimont Z, Andres RJ, Volke A, and Delgado Arias S: Anthropogenic sulfur dioxide emissions: 1850–2005, Atmos. Chem. Phys, 11, 1101–1116, 10.5194/acp-11-1101-2011, 2011.

[R98] Stein AF, Draxler RR, Rolph GD, Stunder BJB, Cohen MD, and Ngan F: NOAA’s HYSPLIT Atmospheric Transport and Dispersion Modeling System, B. Am. Meteor. Soc, 96, 2059–2077, 2015.

[R99] Taylor SR and McLennan SM: The continental crust: Its composition and evolution, Oxford, Blackwell Scientific Publications, ISBN 0632011483, 1985.

[R100] Trapp JM, Millero FJ, and Prospero JM: Trends in the solubility of iron in dust-dominated aerosols in the equatorial Atlantic trade winds: Importance of iron speciation and sources, Geochem. Geophys, 11, Q03014, 10.1029/2009GC002651, 2010.

[R101] Tsamalis C, Chédin A, Pelon J, and Capelle V: The seasonal vertical distribution of the Saharan Air Layer and its modulation by the wind, Atmos. Chem. Phys, 13, 11235–11257, 10.5194/acp-13-11235-2013, 2013.

[R102] Val S, Liousse C, Doumbia EHT, Galy-Lacaux C, Cachier H, Marchand N, Badel A, Gardrat E, Sylvestre A, and Baeza-Squiban A: Physico-chemical characterization of African urban aerosols (Bamako in Mali and Dakar in Senegal) and their toxic effects in human bronchial epithelial cells: Description of a worrying situation, Part. Fibre Toxicol, 10, 1–16, http://www.particleandfibretoxicology.com/content/10/1/10 (last access: 10 July 2024), 2013. 23548138 10.1186/1743-8977-10-10 PMC3637552

[R103] Van der Werf GR, Randerson JT, Collatz GJ, and Giglio L: Carbon emissions from fires in tropical and subtropical ecosystems, Glob. Change Biol, 9, 547–562, 10.1046/J.1365-2486.2003.00604.X, 2003.

[R104] Vannucci PF, Foley K, Murphy BN, Hogrefe C, Cohen RC, and Pye HOT: Temperature-dependent composition of summertime PM_2.5_ in observations and model predictions across the Eastern U.S., ACS Earth Space Chem., 8, 381–392, 2024. 39440258 10.1021/acsearthspacechem.3c00333 PMC11492923

[R105] Vasilakos P, Russell A, Weber R, and Nenes A: Understanding nitrate formation in a world with less sulfate, Atmos. Chem. Phys, 18, 12765–12775, 10.5194/acp-18-12765-2018, 2018.

[R106] Wang S, Maltrud M, Elliott S, Cameron-Smith P, and Jonko A: Influence of dimethyl sulfide on the carbon cycle and biological production, Biogeochemistry, 138, 49–68, 10.1007/s10533-018-0430-5, 2018.

[R107] Wex H, Dieckmann K, Roberts GC, Conrath T, Izaguirre MA, Hartmann S, Herenz P, Schäfer M, Ditas F, Schmeissner T, Henning S, Wehner B, Siebert H, and Stratmann F: Aerosol arriving on the Caribbean island of Barbados: physical properties and origin, Atmos. Chem. Phys, 16, 14107–14130, 10.5194/acp-16-14107-2016, 2016.

[R108] Wiedinmyer C, Akagi SK, Yokelson RJ, Emmons LK, Al-Saadi JA, Orlando JJ, and Soja AJ: The Fire INventory from NCAR (FINN): a high resolution global model to estimate the emissions from open burning, Geosci. Model Dev, 4, 625–641, 10.5194/gmd-4-625-2011, 2011.

[R109] Wolter K and Timlin MS: El Niño/Southern Oscillation behaviour since 1871 as diagnosed in an extended multivariate ENSO index (MEI.ext), Int. J. Climatol, 31, 1074–1087, 10.1002/JOC.2336, 2011.

[R110] Yang Y, Lou S, Wang H, Wang P, and Liao H: Trends and source apportionment of aerosols in Europe during 1980–2018, Atmos. Chem. Phys, 20, 2579–2590, 10.5194/acp-20-2579-2020, 2020.

[R111] Zhang T, Hoell A, Perlwitz J, Eischeid J, Murray D, Hoerling M, and Hamill TM: Towards probabilistic multivariate ENSO monitoring, Geophys. Res. Lett, 46, 10532–10540, 10.1029/2019GL083946, 2019.

[R112] Zhao B, Jiang JH, Gu Y, Diner D, Worden J, Liou KN, Su H, Xing J, Garay M, and Huang L: Decadalscale trends in regional aerosol particle properties and their linkage to emission changes, Environ. Res. Lett, 12, 054021, 10.1088/1748-9326/AA6CB2, 2017.

[R113] Zhao J, Zhang Y, Bie S, Bilsback KR, Pierce JR, and Chen Y: Changes in global DMS production driven by increased CO_2_ levels and its impact on radiative forcing, NPJ Clim. Atmos. Sci, 7, 1–8, 10.1038/s41612-024-00563-y, 2024.

[R114] Zubkova M, Boschetti L, Abatzoglou JT, and Giglio L: Changes in fire activity in Africa from 2002 to 2016 and their potential drivers, Geophys. Res. Lett, 46, 7643–7653, 10.1029/2019GL083469, 2019. 32440032 PMC7241591

[R115] Zuidema P.: Data contributing to Zuidema et al., 2019: Ϊs summer African dust arriving earlier at Barbados? The updated long-term in-situ dust mass concentration time series from Ragged Point, Barbados and Miami, Florida, ^..^ B. Am. Meteorol. Soc. [data set], University of Miami Libraries, 10.17604/q3vf-8m31, 2019.

[R116] Zuidema P, Redemann J, Haywood J, Wood R, Piketh S, Hipondoka M, and Formenti P: Smoke and clouds above the Southeast Atlantic: Upcoming field campaigns probe absorbing aerosol’s impact on climate, B. Am. Meteor. Soc, 97, 1131–1135, 10.1175/BAMS-D-15-00082.1, 2016.

[R117] Zuidema P, Sedlacek AJ, Flynn C, Springston S, Delgadillo R, Zhang J, Aiken AC, Koontz A, and Muradyan P: The Ascension Island boundary layer in the remote Southeast Atlantic is often smoky, Geophys. Res. Lett, 45, 4456–4465, 10.1002/2017GL076926, 2018.

[R118] Zuidema P, Alvarez C, Kramer SJ, Custals L, Izaguirre M, Sealy P, Prospero JM, and Blades E: Is summer African dust arriving earlier to Barbados? The updated long-term in situ dust mass concentration time series from Ragged Point, Barbados, and Miami, Florida, B. Am. Meteor. Soc, 100, 1981–1986, 10.1175/BAMS-D-18-0083T, 2019.

