# Supplementary material for: Diverging trends in aerosol sulfate and nitrate measured in the remote North Atlantic in Barbados are attributed to clean air policies, African smoke, and anthropogenic emissions
Source: Atmos Chem Phys. Author manuscript; Available in PMC 2025 Jul 16. (PMC11534066; doi:10.5194/acp-24-8049-2024)
Supplement: Supplement1 [file NIHMS2021740-supplement-Supplement1.pdf]

Supplement of Atmos. Chem. Phys., 24, 8049–8066, 2024
https://doi.org/10.5194/acp-24-8049-2024-supplement
© Author(s) 2024. CC BY 4.0 License.

*Supplement of*

# Diverging trends in aerosol sulfate and nitrate measured in the remote North Atlantic in Barbados are attributed to clean air policies, African smoke, and anthropogenic emissions

**Cassandra J. Gaston et al.**

*Correspondence to:* Cassandra J. Gaston (cgaston@miami.edu)

The copyright of individual parts of the supplement might differ from the article licence.

**Table S1:** Normalized mean bias (NMB), mean bias (MB), root-mean-sqaure error (RMSE), and the Pearson correlation coefficient (r) for EQUATES model runs for nitrate and nss-sulfate compared to observations for each year from Ragged Point.

| Year | Compound | NMB (%) | MB (ug/m3) | RMSE (ug/m3) | r (pearson correlation coefficient) |
|---|---|---|---|---|---|
| 2002 | Nitrate | -10.18 | -0.06 | 0.19 | -0.22 |
| | NSS-Sulfate | 81.45 | 0.54 | 0.60 | 0.79 |
| 2003 | Nitrate | -6.18 | -0.03 | 0.15 | 0.60 |
| | NSS-Sulfate | 57.70 | 0.39 | 0.51 | 0.65 |
| 2004 | Nitrate | -5.99 | -0.03 | 0.16 | 0.59 |
| | NSS-Sulfate | 41.29 | 0.27 | 0.37 | 0.65 |
| 2005 | Nitrate | -24.18 | -0.15 | 0.24 | 0.84 |
| | NSS-Sulfate | 3.13 | 0.02 | 0.29 | 0.72 |
| 2006 | Nitrate | -17.21 | -0.10 | 0.28 | 0.45 |
| | NSS-Sulfate | 28.15 | 0.21 | 0.42 | 0.38 |
| 2007 | Nitrate | -6.51 | -0.04 | 0.14 | 0.62 |
| | NSS-Sulfate | 40.13 | 0.31 | 0.40 | 0.64 |
| 2008 | Nitrate | -35.06 | -0.26 | 0.35 | 0.52 |
| | NSS-Sulfate | 3.97 | 0.03 | 0.30 | 0.55 |
| 2009 | Nitrate | -9.51 | -0.06 | 0.20 | 0.28 |
| | NSS-Sulfate | 23.89 | 0.16 | 0.35 | 0.23 |
| 2010 | Nitrate | -28.72 | -0.23 | 0.31 | 0.83 |
| | NSS-Sulfate | -14.42 | -0.13 | 0.29 | 0.41 |
| 2011 | Nitrate | -11.57 | -0.07 | 0.12 | 0.88 |
| | NSS-Sulfate | -8.80 | -0.08 | 0.24 | 0.82 |

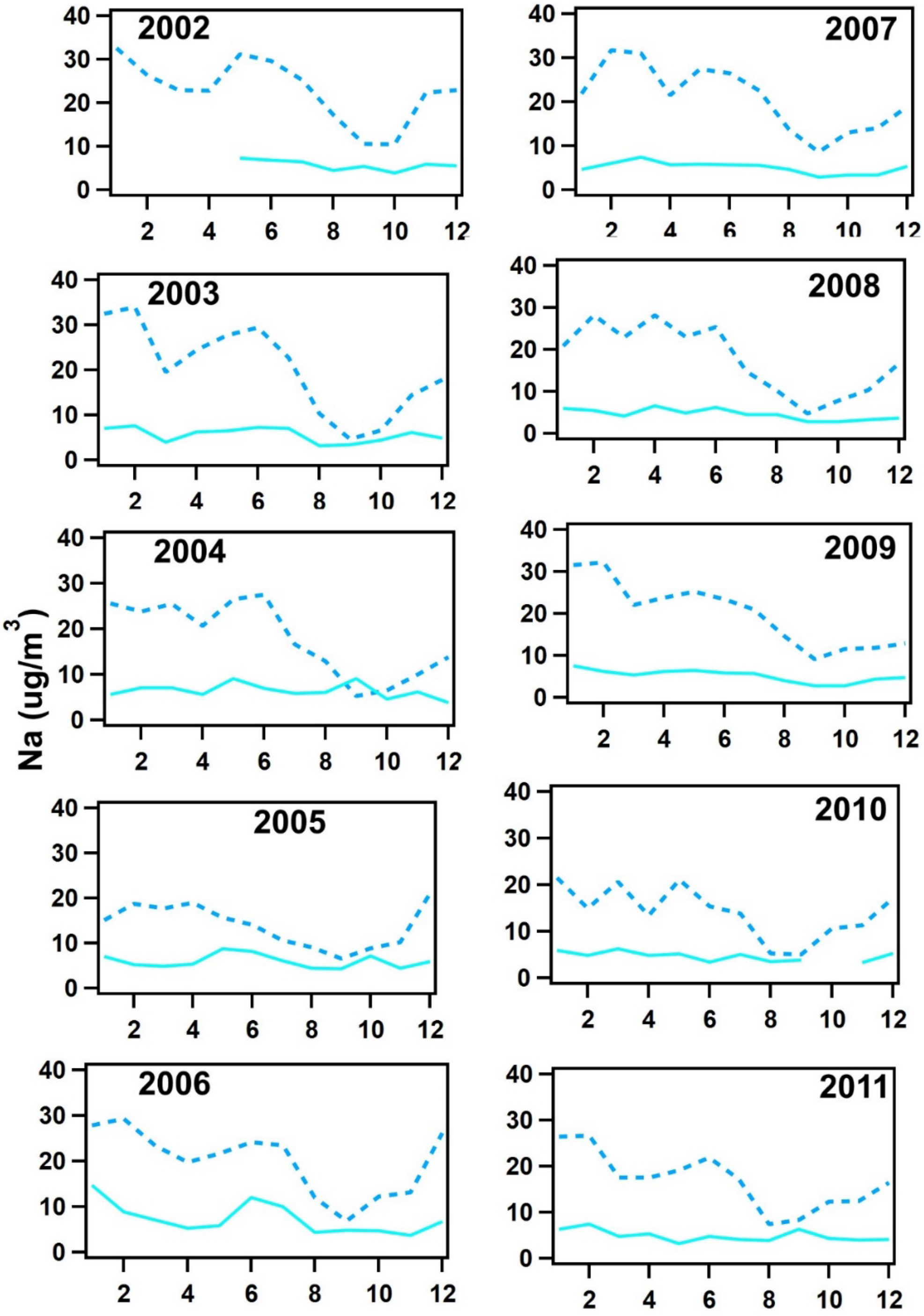


**Figure S1:** Monthly comparison of sodium ($Na^+$) mass concentrations measured at Barbados on filters (solid blue line) and predicted by the EQUATES model (dashed blue line) from 2002-2011.

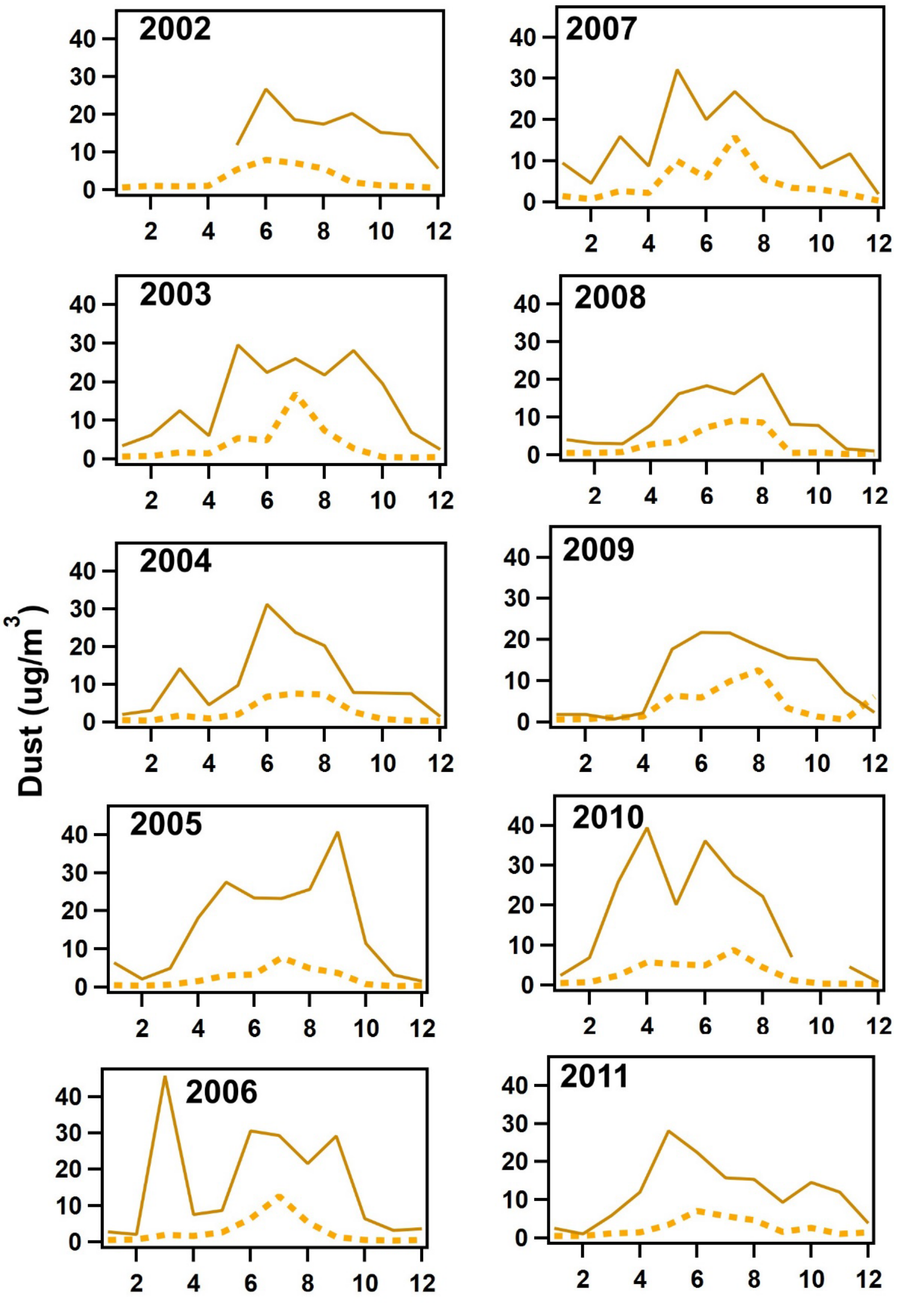


**Figure S2:** Monthly comparison of dust mass concentrations measured at Barbados on filters (solid brown line) and dust predicted by the EQUATES model (dashed brown line) using nss-$Ca^{2+}$ concentrations scaled to the upper crustal abundance of calcium for 2002-2011.

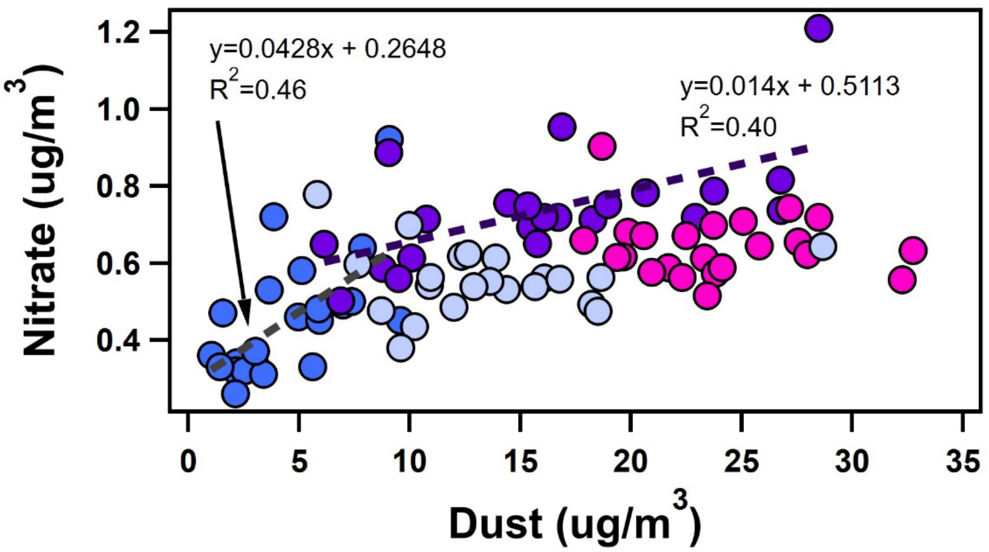


**Figure S3:** Comparison of nitrate and dust mass concentrations as a function of season with DJF in blue, MAM in purple, JJA in pink, and SON in light blue.

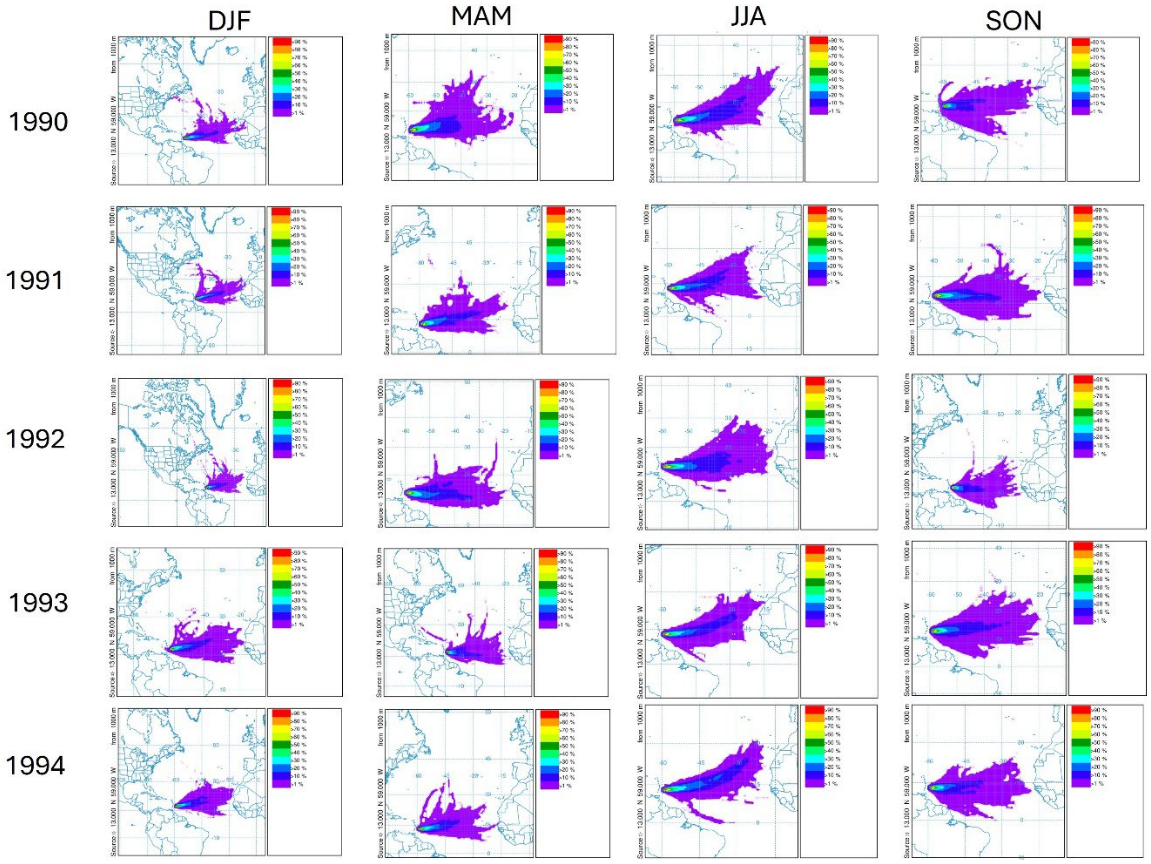

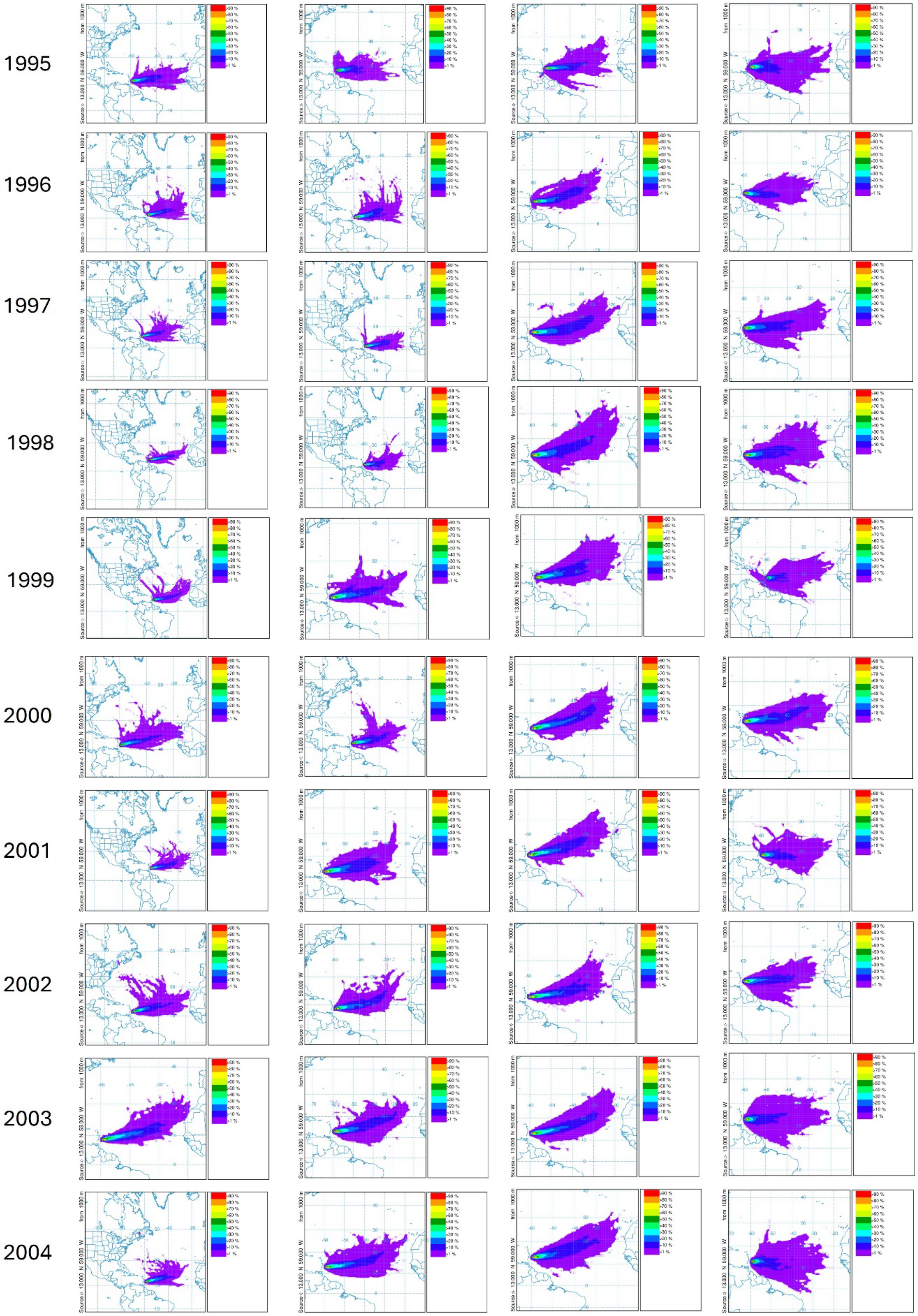
1995
1996
1997
1998
1999
2000
2001
2002
2003
2004

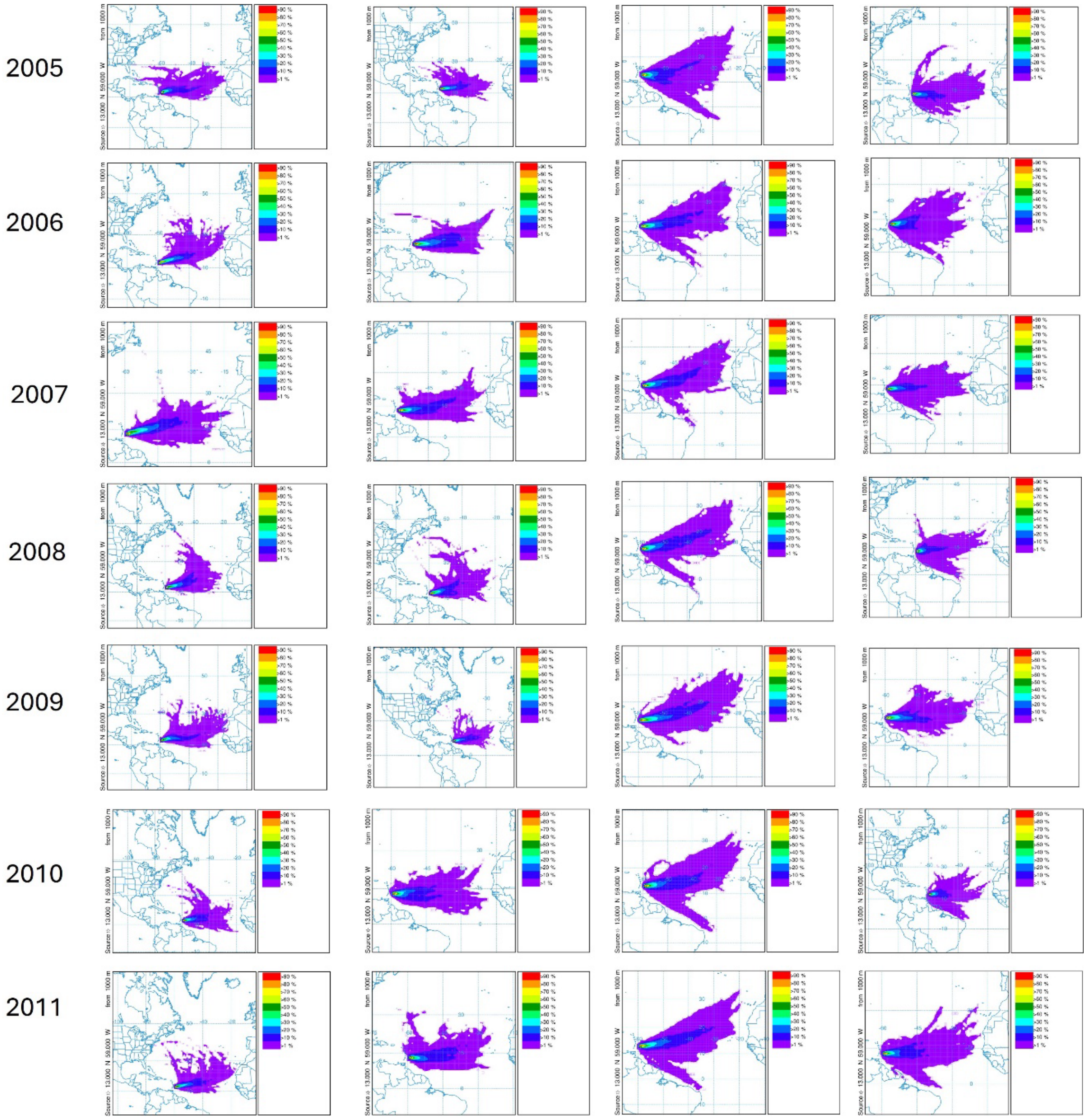


**Figure S4:** Seasonal HYSPLIT back trajectory frequency plots for DJF (Winter), MAM (Spring), JJA (summer), and SON (Fall). 5-day air mass back-trajectories were initiated at a height of 1000 m.

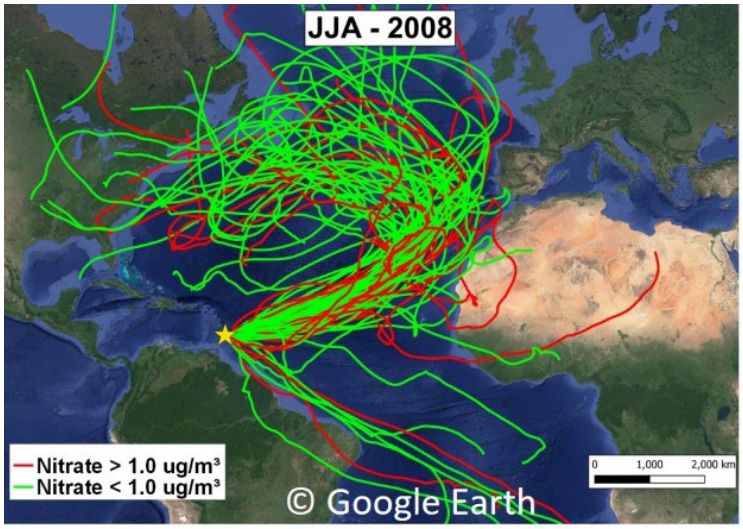


**Figure S5:** 315-h air mass back-trajectories initiated at 500-m for JJA 2008. Back-trajectories labeled in green are for days where nitrate concentrations measured at Ragged Point were < 1 ug/m$^3$ while trajectories labeled in red had nitrate > 1 ug/m$^3$. Maps are from Google Earth (©Google Earth).

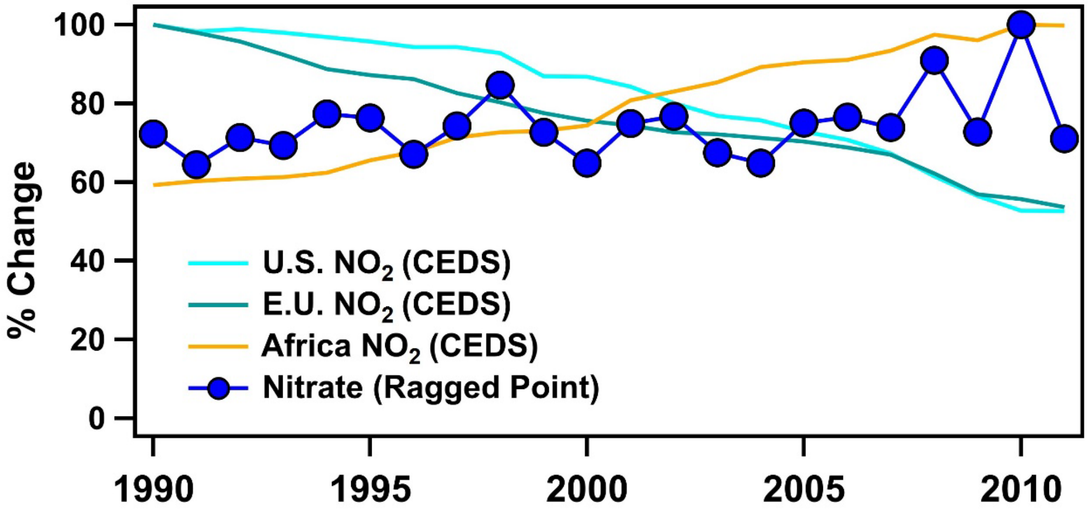


**Figure S6:** Nitrate mass concentrations measured at Ragged Point from 1990-2011 are shown in blue lines with markers. Emissions of nitrogen dioxide ($NO_2$) from McDuffie et al., 2020 are included for comparison. Decreasing emissions of $NO_2$ from the US and EU are shown in blue lines. Increasing emissions of $NO_2$ from Africa are shown in orange.

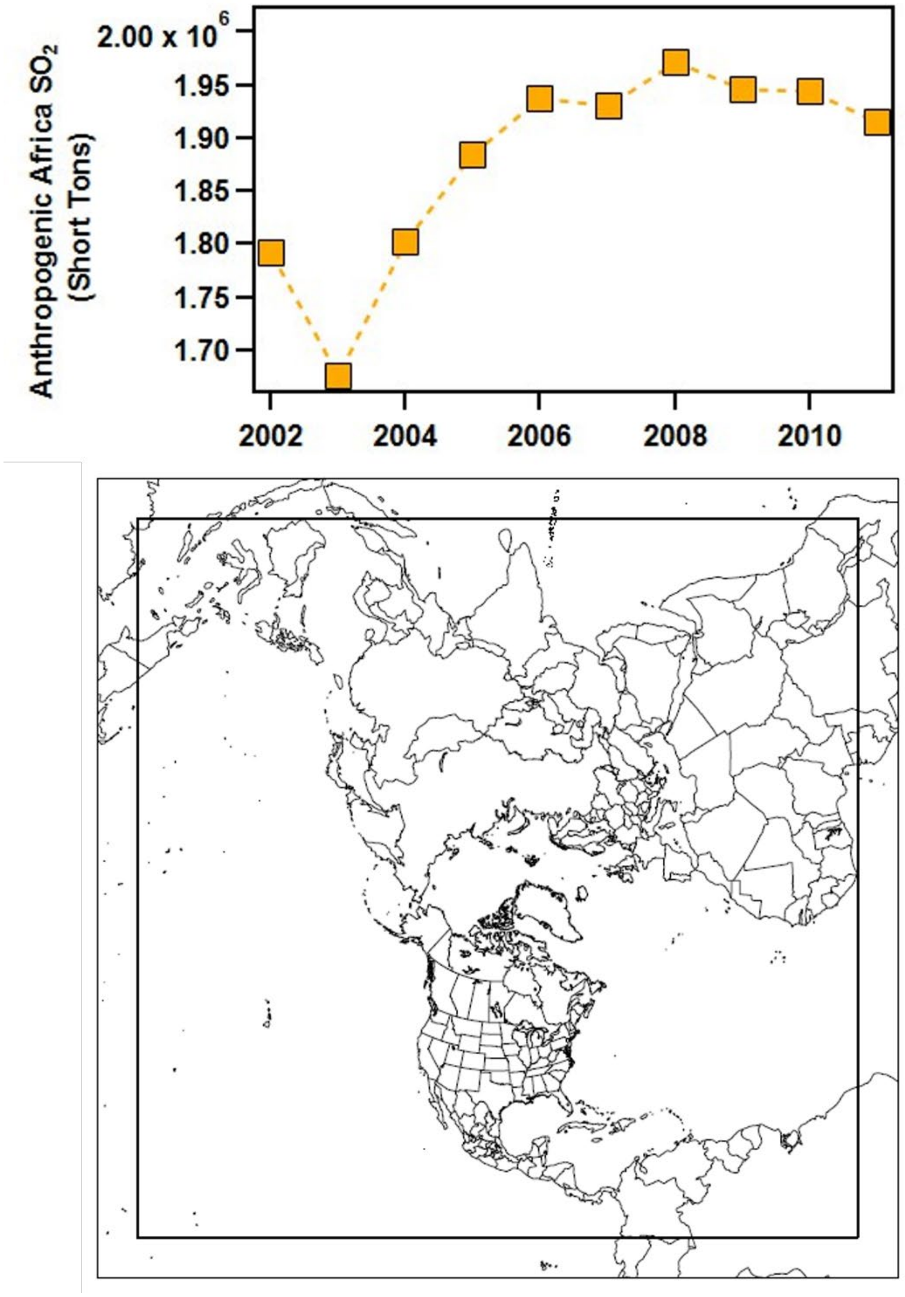


**Figure S7:** EQUATES simulations of $SO_2$ emissions from Africa from anthropogenic sources from 2002-2011 (top panel). Note that only part of Africa is included in the EQUATES Northern Hemisphere domain as shown in the bottom panel, thus, emissions do not reflect the entire continent. The bottom panel shows the EQUATES domain over the Northern Hemisphere (187 rows x 187 columns x 44 vertical layers). Horizontal grid resolution is 108 km x 108 km.

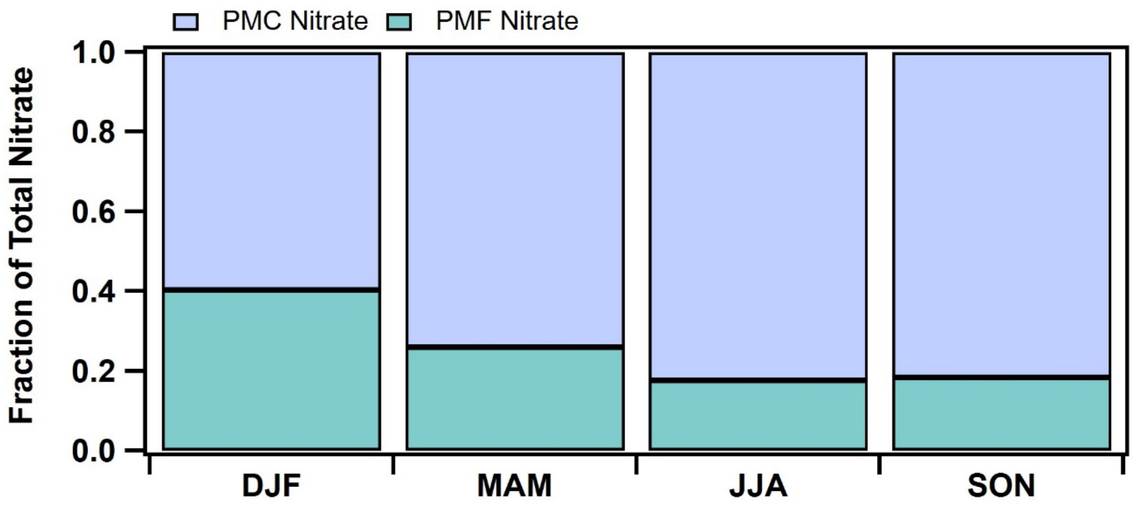


**Figure S8:** Seasonal averages of coarse mode (PMC, light blue) and fine mode (PMF, dark teal) contributions to aerosol nitrate predicted by EQUATES.

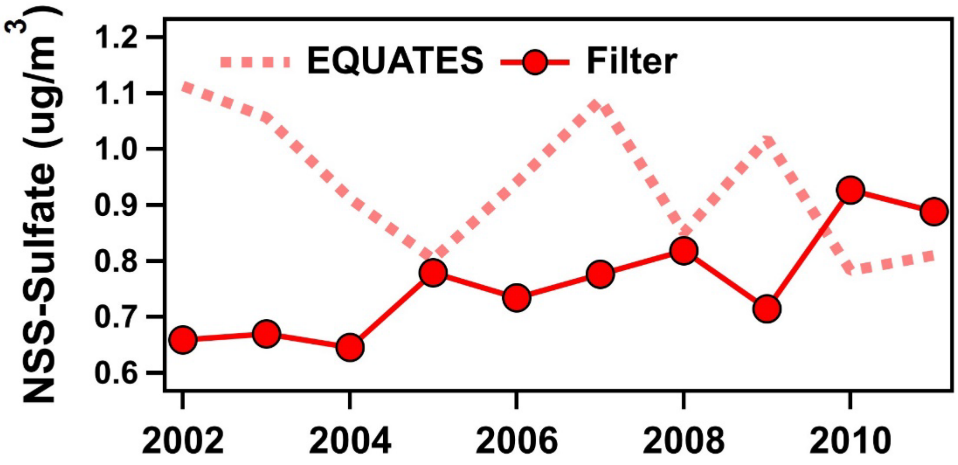


**Figure S9:** Annual averages of NSS-Sulfate measured from filters collected at Ragged Point (solid red line) and predicted by EQUATES (dashed pink line).

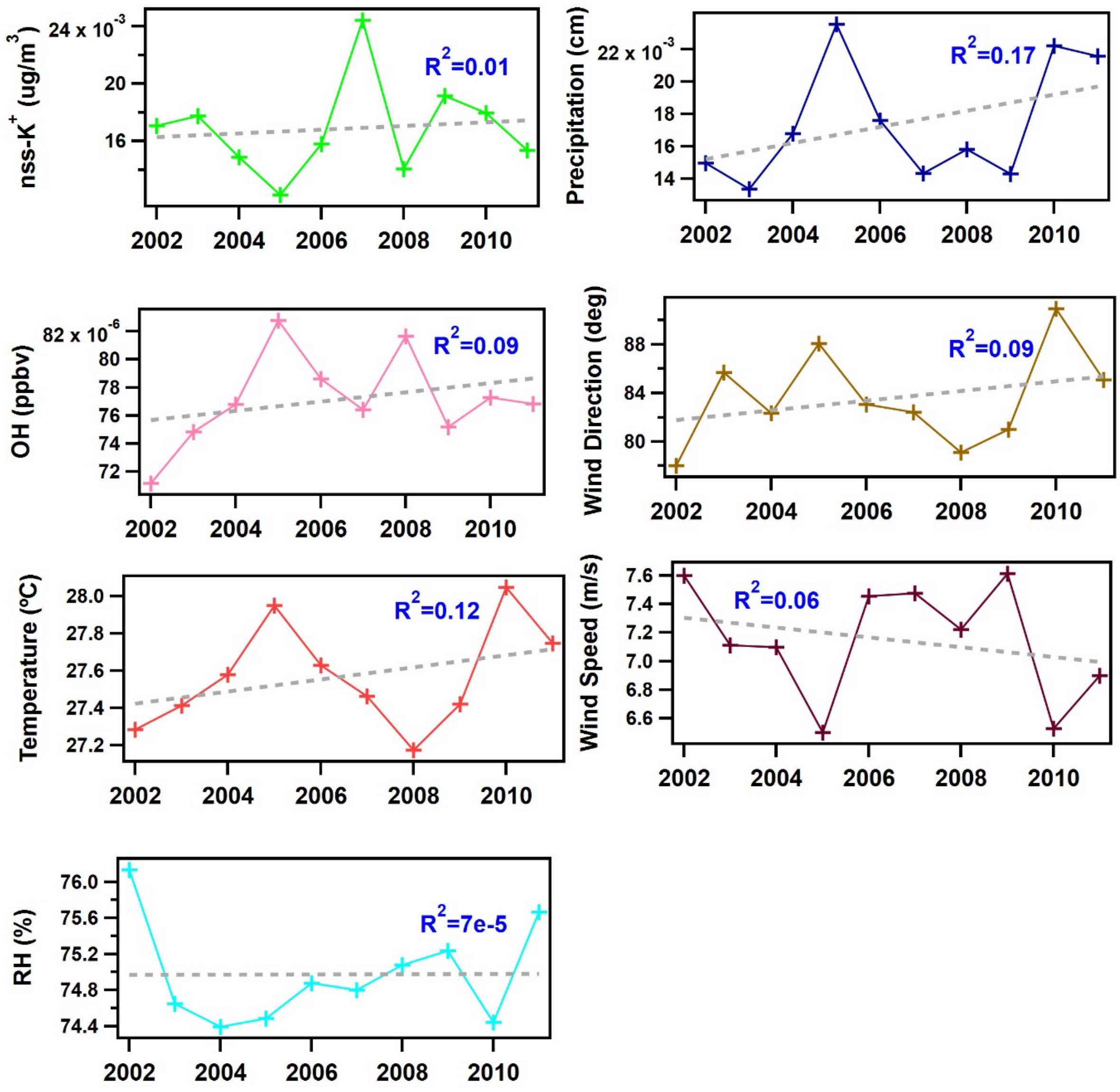


**Figure S10:** Annual averages of non-sea salt-potassium (nss-K+), hydroxyl radical concentrations (OH), temperature, relative humidity (RH), wind speed, and wind direction calculated from EQUATES model predictions. Linear fits and correlation coefficients are also shown.
